# Adolescent Cultural Identity Development in Context: The Dynamic Interplay of the Identity Project With Classroom Cultural Diversity Climate in Italy and Germany

**DOI:** 10.1007/s10964-024-02031-5

**Published:** 2024-06-28

**Authors:** Maja K. Schachner, Sophie Hölscher, Ughetta Moscardino, Chiara Ceccon, Linda Juang, Massimiliano Pastore

**Affiliations:** 1 Martin Luther University Halle-Wittenberg, Halle, Germany; 2 University of Padova, Padua, Italy; 3 University of Potsdam, Potsdam, Germany

**Keywords:** Identity Project, Classroom cultural diversity climate, Cultural identity development, Intervention, School

## Abstract

While both the classroom cultural diversity climate and curriculum-based interventions can promote cultural identity development, they have not been studied together. Drawing on theories of ethnic-racial identity development, the current study aimed to understand the dynamic interplay of a curriculum-based intervention (the Identity Project) with the classroom cultural diversity climate (heritage culture and intercultural learning, critical consciousness socialization and equal treatment) on cultural identity exploration and resolution. Our sample included 906 mid-adolescents in Italy (32.36% immigrant descent, *M*_age_ (*SD*) = 15.12 (0.68) years, 51.73% female), and 504 early adolescents in Germany (53.86% immigrant descent, *M*_age_ (*SD*) = 12.82 (0.89) years, 42.37% female). Bayesian multivariate linear models show that the Identity Project and a stronger critical consciousness climate in the classroom before the intervention promoted cultural identity exploration at post-test in both countries. However, effects of the intervention and facets of the diversity climate on subsequent resolution were only observed in Italy. There was some evidence that the intervention could alter the classroom cultural diversity climate in Germany, while the intervention could compensate for a less positive diversity climate in the slightly older sample in Italy. Thus, it seems promising to systematically build in opportunities to engage with students’ diverse heritage cultures and identities when developing new curricula, as well as to train teachers to implement such curricula.

## Introduction

Adolescents who have gained clarity and feel positive about their ethnic-racial identity, in Europe often conceptualized as (heritage) cultural identity 1 (Juang et al., 2022), experience higher self-esteem, fewer mental health problems, and do better academically, and these effects are especially pronounced amongst youth from marginalized ethnic-racial groups (Umaña-Taylor & Rivas-Drake, 2021). The Identity Project is a school-based intervention designed in the United States to promote ethnic-racial identity development, thereby contributing to positive developmental outcomes (Umaña-Taylor, Douglass, et al., 2018). One of the original goals of the intervention was to promote a classroom climate that respects and values diversity (Umaña-Taylor & Douglass, 2017). Yet, this has not been the focus of initial efficacy studies. At the same time, efficacy of the intervention curriculum may vary depending on the cultural diversity climate in the classrooms where it is implemented. While most of the studies on ethnic-racial identity development have been conducted in the United States, the current study investigates the dynamic interplay of the Identity Project with the classroom cultural diversity climate in secondary schools in Italy and Germany, both of which are major countries of immigration in Europe.

### Ethnic-Racial Identity Development in Adolescence

Developing a cohesive identity is a core developmental task in adolescence (Erikson, 1968), with consequences for psychological well-being and academic adjustment (Crocetti, 2017). In increasingly diverse societies, ethnic-racial identity, defined as “the beliefs and attitudes that individuals have about their ethnic–racial group memberships, as well as the processes by which these beliefs and attitudes develop over time” (Umaña-Taylor et al., 2014, p. 23), is an important facet of adolescent identity. Ethnic-racial identity is developed through exploration (i.e., engaging with one’s ethnic-racial background and trying to learn more about it), which may then lead to resolution (i.e. gaining clarity about the personal meaning of one’s background) (Umaña-Taylor et al., 2014). The latter is sometimes also referred to as commitment (Umaña-Taylor et al., 2004), following earlier conceptualizations of ethnic-racial identity development (Phinney, 1992). Exploration is particularly salient in adolescence and may increase from middle adolescence with more independence from parents and resistance to peer pressure (Steinberg & Monahan, 2007; Umaña-Taylor et al., 2004). However, in highly diverse contexts with frequent interethnic contact, and following school transitions in early adolescence, it is likely that youth already engage in exploration processes earlier (García Coll et al., 1996). Indeed, a study with Mexican-American youth in the United States showed increasing ethnic-racial identity exploration already in early adolescence (Matsunaga et al., 2010).Ethnic-racial identity, in Europe often studied through an acculturation framework (Erentaitė et al., 2018), develops through social interactions. It is therefore heavily dependent on context, and to what extent the context is experienced as supportive or stressful (Suárez-Orozco et al., 2018; Umaña-Taylor et al., 2014). School forms an important context during adolescence, as youth become more oriented outside of the family and spend more time at school (Eccles & Roeser, 2011). School is also an important context for acculturation and intercultural contact (Schachner et al., 2018). Both the broader classroom climate (e.g., to what extent norms and practices are experienced as positive and supportive of adolescents’ needs), and the curriculum (e.g., to what extent the topics and materials are meaningful and responding to adolescents’ experiences and developmental needs), have implications for acculturation-related processes and adolescent development more broadly, including ethnic-racial or cultural identity development (Eccles & Roeser, 2011; Schachner et al., 2018). The broader societal context is also relevant and can shape and interact with how diversity is addressed at school (Suárez-Orozco et al., 2018): The societal context may vary, for example, in terms of the perception by policymakers and the general public of being or not being a country of immigration. These perceptions may translate into more or less favorable immigration and diversity policies and practices, but also inform concepts of national identity, and discussions around citizenship and multiculturalism. When perceived as a country of immigration, the government may be more likely, for instance, to engage in policy efforts to support immigrant integration and participation in communities and institutions.

### Classroom Cultural Diversity Climate and Ethnic-Racial or Heritage Cultural Identity

Different facets of the school or classroom cultural diversity climate have been linked to ethnic-racial or heritage cultural identity (Moscardino et al., 2023; Schachner et al., 2016). In this study, we focus on (1) heritage culture and intercultural learning, often subsumed under a multicultural or pluralism approach, (2) a more recently added dimension of critical consciousness socialization (Schachner et al., 2021), and (3) equal treatment. We chose these facets both for their theoretical relevance for identity development in terms of cultural socialization (Berry & Georgas, 2009) and rejection-identification (Branscombe et al., 1999) processes as well as on the basis of previous empirical evidence. Rooted in culturally responsive teaching and multicultural education (Banks, 1993; Gay, 1975), a pluralism approach means cultural diversity is acknowledged, valued, seen as a resource, and opportunities are provided to learn about this diversity and the diverse heritage cultures of students. Critical consciousness socialization on the other hand captures to what extent there are also discussions about social inequality and systemic racism in society and how to address this (Byrd, 2017; Schachner et al., 2021). Finally, equal treatment draws on intergroup contact research (Allport, 1954; Pettigrew, 1998) and taps into norms and practices of equality and the absence of discrimination in the classroom.

#### Heritage culture and intercultural learning

Drawing on models of cultural socialization (Berry & Georgas, 2009), heritage culture and intercultural learning can promote both enculturation (socialization in one’s own culture) and acculturation (socialization in another culture), which are in turn connected to identity development (Crocetti et al., 2024). For example, when engaging with cultural norms and traditions in students’ families, they get an opportunity to explore their own family’s cultural background but also learn something about the cultural backgrounds of other students in the classroom, both of which may help them in their personal and cultural identity development. In support of this, more support for heritage culture and intercultural learning has been connected longitudinally to stronger heritage cultural identity (Schachner et al., 2016) and more stable heritage cultural identity trajectories (Juang et al., 2023) amongst early adolescents of immigrant descent in Germany. Similarly, more support for pluralism in class was associated cross-sectionally with stronger heritage cultural identity amongst early and mid-adolescents of immigrant descent in Germany (Aral et al., 2023). Longitudinal and cross-sectional research from Italy and the US shows that early and mid-adolescents of immigrant and non-immigrant descent perceiving more support for cultural pluralism at school showed greater ethnic-racial and heritage cultural identity exploration and resolution (Camacho et al., 2018; Moscardino et al., 2023).

#### Critical consciousness socialization

Critical consciousness socialization has only recently been added to measures of the perceived cultural diversity climate in the United States (Byrd, 2017) and in Germany (Schachner et al., 2021). It taps into creating awareness of discrimination and to what extent there is discussion acknowledging discrimination in school and society and has been associated with individual discrimination experiences (Byrd, 2017; Schachner et al., 2021). Thus, it may trigger rejection-identification processes (Branscombe et al., 1999), where experiencing rejection from an out-group strengthens one’s identification with an in-group. Research on individual critical consciousness development suggests a reciprocal relation with ethnic-racial identity development (Mathews et al., 2020). For example, when adolescents reflect about societal inequities, with some groups experiencing more privilege or disadvantage than others, they may also be inclined to explore about their own group and what it means to be a member of this group in their society. Reciprocal and cross-sectional associations between ethnic-racial identity and individual critical consciousness were confirmed empirically amongst mid- and late adolescents in the US (Kiang et al., 2021; Mathews et al., 2022). Based on theoretical frameworks of individual development in context (e.g., Suárez-Orozco et al., 2018) and empirical evidence concerning effects of other facets of the classroom cultural diversity climate on ethnic-racial identity development reported above, a directional effect of critical consciousness socialization in the classroom on individual cultural identity development will be tested.

#### Equal treatment

It seems plausible that adolescents experiencing more equal treatment at school may feel more open to explore, gain clarity, and feel a sense of belonging to their heritage culture. Yet, research shows either no relation (Juang et al., 2023; Schachner et al., 2016) or a negative relation (Aral et al., 2023) of equal treatment climate with heritage cultural identity. Equal treatment is sometimes equated with color-evasion, where diversity is consciously neglected and ignored, and this may partly explain the absent or negative associations with heritage cultural identity. Another explanation could be that equal treatment is often (also in the current study) measured with reverse-items, which do not indicate equality but the absence of inequality. In line with rejection-identification hypothesis (Branscombe et al., 1999), early adolescents of immigrant descent in Germany who experienced more ethnic-racial discrimination and inequality also reported stronger heritage cultural identities (Fleischmann et al., 2019). Thus, the absence of inequality might produce the reverse effect, i.e. weaker heritage culture identities. Nevertheless, given the conflicting evidence from previous studies, we will investigate effects of equal treatment in an exploratory fashion.

### The Identity Project and its Interplay with the Classroom Cultural Diversity Climate

The Identity Project is a school-based intervention specifically designed to support adolescents in their ethnic-racial identity development and to promote a classroom climate that respects and values diversity (Umaña-Taylor & Douglass, 2017). The Identity Project comprises a detailed curriculum for eight sessions, including a wide range of activities and materials to engage with ethnic, racial, and cultural diversity, taking a critical perspective and looking at historical and contemporary issues. Adolescents in the US who participated in the intervention engaged in more ethnic–racial identity exploration, which promoted higher ethnic–racial and global identity resolution one year later (Umaña-Taylor, Douglass, et al., 2018). This was then associated with a wide range of positive outcomes, including better academic achievement, socio-emotional adjustment and intergroup attitudes one year later (Umaña-Taylor, Kornienko, et al., 2018). Engaging with ethnic, racial, and cultural diversity in class as part of the intervention curriculum starts a cascading effect of ethnic-racial identity development that ultimately translates into better adjustment, as outlined in a theory of change (Umaña-Taylor, 2023). There is also initial evidence for effects on heritage cultural identity exploration in Germany and from a randomized control trial using the same intervention in Italy (Ceccon et al., 2023; Juang et al., 2020).

#### The Identity Project as an intervention to improve classroom cultural diversity climate

While the Identity Project curriculum was developed with the aim to influence the classroom climate (Umaña-Taylor & Douglass, 2017), changes in the perceived diversity climate in schools as a result of the intervention have not been the focus of initial efficacy studies. Yet, a study of US college students identified the inclusion of diversity-related topics into the curriculum as a core predictor of students’ diversity climate perceptions (Mayhew et al., 2005). There is also evidence from Germany that adolescents perceived more unequal treatment and a stronger critical consciousness climate in their classroom after having gone through the Identity Project curriculum (Juang et al., 2020). This suggests that adolescents are more sensitive to discrimination happening in their immediate environment at school as a result of having engaged with ethnic, racial, and cultural diversity as part of the curriculum, and they also perceive more discussions and awareness of systemic racism and inequality in society in their classroom. As the intervention is about engaging with and exploring students’ diverse heritage cultures, we also expect an effect on perceived heritage culture and intercultural learning climate. Since all three facets of the classroom cultural diversity climate are likely associated with cultural identity as outlined above, changes in the climate after the intervention may further promote cultural identity development at later time points.

#### Classroom cultural diversity climate altering intervention effects on identity development

Finally, the cultural diversity climate may moderate intervention effects. The Identity Project is considered a universal intervention promoting ethnic-racial identity development and socio-emotional adjustment for all youth. Yet, person-environment fit theory (Eccles et al., 1993) proposes that for positive development to occur, there needs to be a match between adolescents’ developmental needs and the opportunities the context provides for such development to take place. Similarly, psychological affordances in context (Walton & Yeager, 2020) suggests that for an intervention to be effective, the context where it is delivered should afford the way of thinking inherent in the intervention. It is therefore likely that there is variation in intervention efficacy based on contextual conditions outside of the intervention, and the extent to which these are congruent with the developmental processes it aims to promote, as well as the intervention content and perspective. Specifically, adolescents who also experience support for and opportunities to engage with their heritage cultural identity outside of the intervention may engage more with the intervention content, resulting in different experiences during and after the intervention. In support of this, effects of the Identity Project on ethnic-racial identity development were stronger for youth experiencing a higher level of ethnic-racial socialization in their family, thereby suggesting an additive effect of context and intervention (Sladek et al., 2021). It seems likely that adolescents experiencing more heritage culture and intercultural learning and critical consciousness in their classroom before and directly after going through the intervention curriculum will engage in more heritage cultural identity exploration and experience a higher degree of resolution as a result of the intervention.

### Studying Cultural Identity Development in Italian and German Secondary Schools

Italy and Germany are major countries of immigration in Europe. Yet, given the different historical context in Europe, especially in countries that were involved in the holocaust like Germany or Italy, the term race and categorizing people into racial groups is considered taboo (Juang et al., 2021). Other labels and constructs are used in public discourse instead, such as heritage culture or cultural background. Often these are used in a racialized way, implicitly still referring to those considered visibly different from an imagined national majority who are non-immigrant, Christian, white (e.g., People of Color, women wearing headscarves). In official statistics, minoritized and majoritized groups are often simply distinguished based on being or not being of immigrant descent, defined as either having a direct migration experience or one or both of the parents having immigrated to the country of residence. However, in public discourse it is again mostly people who are considered visibly different who are perceived as being of immigrant descent, and who experience most marginalization. Ethnicity is therefore strongly connected to visible differences but also having roots in a particular country, which was also the basis for citizenship in countries like Germany until recently, connecting ethnicity to nationality or national identity. The notion of ethnic-racial vs. (heritage) cultural identity and the specific historical context also informed the adaptation of the Identity Project materials for implementation in Italy and Germany and subsequently other European countries (Juang et al., 2022).

Both Italy and Germany considered themselves to be culturally homogenous, coupled with assimilation norms, for a long time, despite turning into countries of immigration starting from the 1950s (Germany) and 1970s (Italy). Assimilation norms are still prevalent in both countries, and the desire of adolescents of immigrant descent to follow an integration strategy is often met by assimilation expectations in the native population, also in the domain of school (for evidence from Italy, see Mancini & Bottura, 2014). As a consequence, evidence from Germany suggests that youth assimilating to what is perceived to be a German mainstream culture fared better in terms of educational attainment than those following an integration strategy, while the opposite pattern emerged for well-being outcomes (Schotte et al., 2018). Assimilation expectations are also reflected in rather unsupportive policies towards immigrants and ethnic minorities, with Italy scoring 1.5 and Germany scoring slightly better, but still only 3 out of 8 on the Multiculturalism Policy Index (2021). Italy is a major entry and transit country into the European Union, while Germany is one of the most popular destination countries in Europe. In Italy, roughly 9% of the population are legally residing citizens of immigrant descent (ISTAT, 2023), with the main heritage groups from Romania, Albania, Morocco, China, and Ukraine. In Germany, 24% of the population is of immigrant descent (i.e. with own migration experience or at least one immigrant parent; Statistisches Bundesamt, 2022). The largest heritage groups are from Turkey, Poland, and Russia. Considering the prevalence of monocultural, assimilationist norms in both countries, it may be even more important for intervention efficacy to consider the moderating effect of the perceived cultural diversity climate in the classroom, which can provide a more supportive micro-context.

## The Present Study

**Fig. 1:**
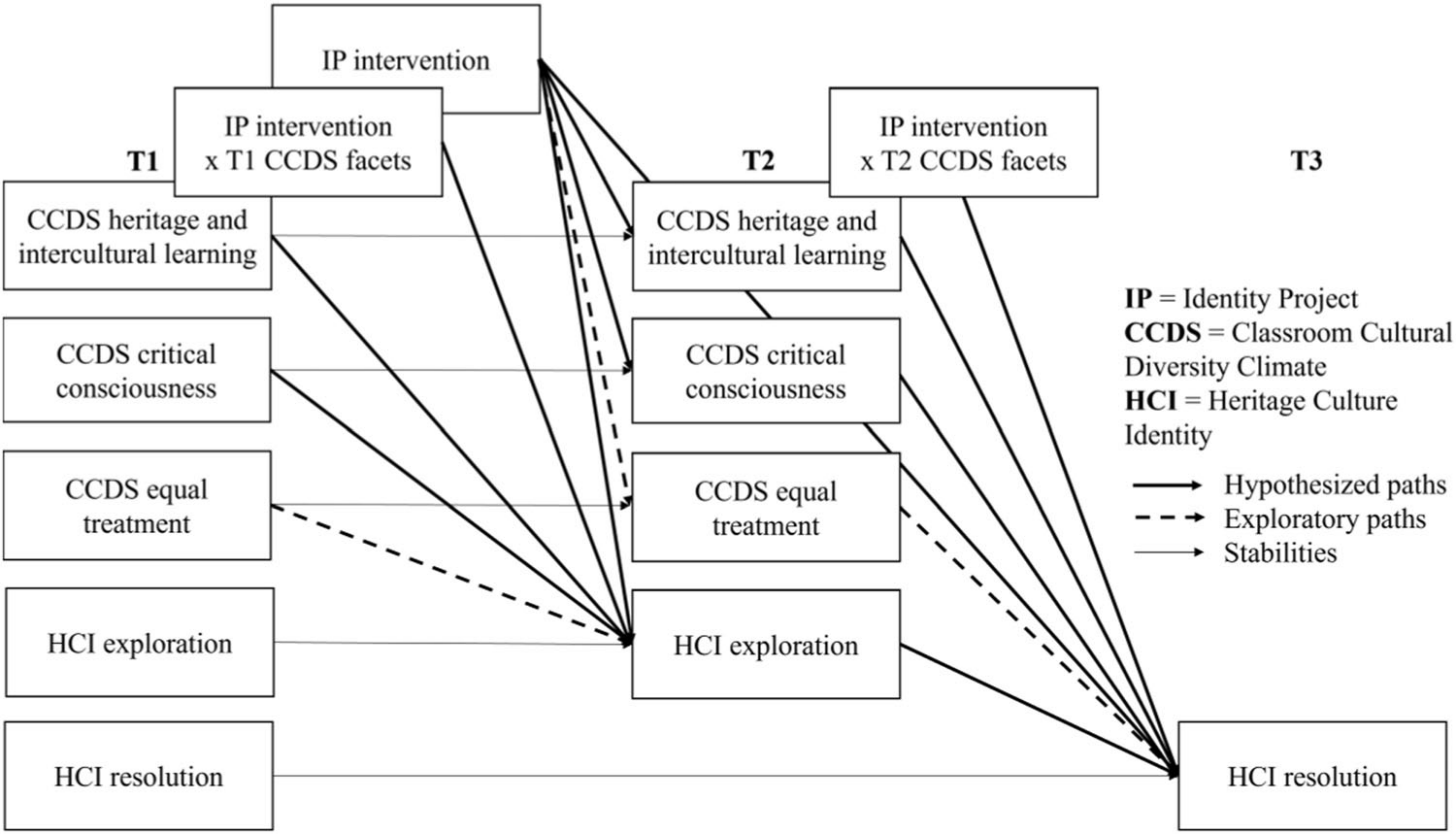
Conceptual model

While both the classroom cultural diversity climate and curriculum-based interventions can promote cultural identity development, they have not been studied together. Drawing on a cascading model and theory of change of ethnic-racial identity development (Umaña-Taylor, 2023) the current study aimed to understand the dynamic interplay of a curriculum-based intervention with the classroom cultural diversity climate on cultural identity development (see conceptual model in Fig. 1). We first tested these associations in a mid-adolescent sample in Italy, and then in a slightly younger sample of early adolescents in Germany. Concerning main effects of the Identity Project and climate on identity (Research Question 1), we expected that adolescents in the intervention group (vs. control group; Hypothesis1a) and those experiencing a more positive cultural diversity climate (more heritage and intercultural learning, more critical consciousness; Hypothesis 1b) at pretest (T1) and post-test (T2) would show increased heritage cultural identity exploration at T2 and resolution at follow-up (T3), directly and indirectly through exploration at T2, and controlling for exploration and resolution at T1. Concerning effects of the Identity Project on climate (Research Question 2), we expected that adolescents in the intervention group (vs. control group) experience a more positive cultural diversity climate (more heritage and intercultural learning, more critical consciousness) at T2, controlling for climate at T1 (Hypothesis 2). Concerning climate dimensions moderating intervention effects on identity (Research Question 3), we expected that the main effect of the intervention on heritage cultural identity exploration at T2 and resolution at T3 would be larger for adolescents who experience a more positive cultural diversity climate (more heritage and intercultural learning, more critical consciousness) at T1 and T2. We also include equal treatment in the analyses as a third dimension of classroom cultural diversity climate in relation to Research Questions 1–3. Yet, as previous research suggests that effects can go in different ways, ranging from no relation (Juang et al., 2023; Schachner et al., 2016) to a negative relation (Aral et al., 2023), we will look at this dimension in an exploratory manner. Our hypotheses, exploratory research question and analysis plan were preregistered (https://osf.io/43tq8/?view_only=13a7f2196b7441f19eb4644b19947453).

## Method

### Intervention Design and Data Collection Procedure

The Identity Project was adapted to the Italian and German context prior to implementation (for a detailed review, see Ceccon, Schachner, Umaña-Taylor, et al., 2023; Juang et al., 2022). The study was designed as a randomized controlled trial at the classroom level. Students from classrooms in the intervention group received the 8-week Identity Project intervention, whereas their peers from classrooms in the control group were put on a waitlist prior to receiving the intervention. A questionnaire was distributed to the intervention and control group 1 week prior to implementing the intervention in the intervention group (T1 – pretest), as well as 1 week after implementing the intervention in the intervention group (T2 – posttest), and 5 weeks (Italy)/8 weeks (Germany) after the intervention and before implementing the intervention in the waitlist control group (T3 – follow-up). In Germany, students were given small gifts (e.g., erasers or chocolate) as a reward, while in Italy due to Covid-related hygiene precautions, no such rewards could be provided. Both the intervention sessions and data collection were carried out in 55 min (Italy) and 90 min (Germany) slots of regular class time. The intervention sessions were implemented by a team of researchers and trained facilitators external to the schools in both countries, with facilitators working in pairs and the same facilitator pair implementing all eight sessions in a classroom. In Germany, facilitator pairs always included one person of immigrant descent and one person of non-immigrant descent, if possible also representing different genders, whereas facilitator pairs in Italy were all female and with the exception of one facilitator all of non-immigrant descent. In Italy, all procedures were approved by the Ethics Committee of the School of Psychology at the University of Padova (protocol n. 3871). In Germany, ethical approval was received from the Berlin Education Senate, as well as the State Board of Education (Landesschulamt) of Saxony-Anhalt.

Schools with a high percentage of non-Italian/non-German first language students were targeted for the study and approached. After establishing informal contacts with schools, the Identity Project was presented and approval was obtained from school principals and teachers, and consent obtained from parents in participating 10^th^ grade (Italy) and 7^th^ grade (Germany) classrooms. In Italy, students whose parents did not provide consent to participate in the project did not take part in the sessions or the survey. In Germany, all students of participating classrooms took part in the project as it was part of the regular curriculum; however, students whose parents denied consent for participation in the accompanying research study did not fill in the survey.

### Participants

In Italy, participants were recruited from 45 classes at in six public upper secondary schools in the Northeast, specifically in the Veneto region, which hosts a large proportion of legally residing citizens of immigrant descent (ISTAT, 2023). Our sample included classes in academic (*n* = 6 classes), technical (*n* = 17 classes), and vocational (*n* = 22 classes) school tracks. The percentage of students of immigrant descent per class ranged from 5.00% to 67.00%.

The Italian sample consisted of *N* = 955 10^th^ grade students (32.36% of immigrant descent, *M*_age_ = 15.12 years, *SD*_age_ = 0.68, 51.73% self-identifying as female, 2.62% self-identifying as non-binary). Among students of immigrant descent, 71.43% were 2^nd^ generation youth (i.e., born in Italy from at least one parent born abroad), while the others were 1st generation youth (i.e., born abroad and then migrated to Italy) who had lived in Italy for an average of 8.63% years (range = 2–16 years). The most common heritage cultures students identified with were Moroccan (12.66%), Albanian (7.14%), Tunisian (3.57%), Chinese (3.25%), and Nigerian (2.60%). Regarding their primary language, 55.51% students of immigrant descent reported speaking Italian, 43.51% reported speaking a language other than Italian, and 1% reported speaking both Italian and another language. The most common languages spoken next to Italian were Arabic (7.79%), Romanian (7.14%), Moldovan (3.90%), Chinese (3.25%), and Albanian (2.60%).

In Germany, participants were recruited from 26 classes at six public secondary schools. This included multiple cohorts from one school in Berlin, with four 7^th^ grade classes participating in the academic year 2018–2019 (*n* = 101), four 7^th^ grade classes participating in the academic year 2019–2020 (*n* = 96), and two 7^th^ grade classes participating in the academic year 2021–2022 (*n* = 26). Additionally, participants were recruited from sixteen 7^th^ grade classes from five high schools in Halle (Saale) in the academic year 2021–2022 (*n* = 360). Our sample included classes in the academic track preparing students for university entrance (*n* = 8 classes), vocational track secondary school classes that do not allow access to university (*n* = 7 classes), mixed track classes (*n* = 10 classes) and one preparatory class, often designed for students who require additional support with the German language before entering regular classes, particularly children with refugee experiences (*n* = 1 class). The percentage of students of immigrant descent per class ranged from 10.71% to 100.00%.

The German sample was slightly younger compared to the Italian sample, with *N* = 583 7^th^ grade students (53.86% of immigrant descent, *M*_age_ = 12.82 years, *SD*_age_ = 0.89, 42.37% self-identifying as female, 1.20% self-identifying as non-binary). Among students of immigrant descent, 56.74% were 2^nd^ generation youth (i.e., born in Germany from at least one parent born abroad), while the others were 1^st^ generation youth (i.e., born abroad and then migrated to Germany) who migrated to Germany at an average age of 7.65 years (*SD*_age_ = 3.28, range = 0–14 years). The most common heritage cultures students identified with were German (34.33%), Turkish (9.20%), and Syrian (4.48%). Regarding primary language spoken with parents, 45.01% of students indicated speaking German, 18.59% indicated speaking a language other than German, and 36.40% indicated speaking both German and another language with their parents. The most common languages spoken next to German were Arabic (10.76%), Turkish (7.24%), and Kurdish (7.05%).

### Measures

Students were asked about their age, gender, birthplace, parents’ birthplace, and heritage culture identification. Consistent with previous research (e.g., Schachner et al., 2016), immigrant descent was coded as 0 (born in Italy/Germany from Italian-born/German-born parents) or 1 (born abroad or in Italy/Germany from at least one parent born abroad). Socioeconomic status (SES) was measured with the 4-item family affluence scale (Currie et al., 2004; e.g., “Does your family have a car?”). Item scores are summed to yield a total score ranging from 0 (lowest affluence) to 9 (highest affluence). For the reliabilities, the range in omega across the three time points is provided.

#### Heritage cultural identity

In Italy, the Ethnic Identity Scale (Umaña-Taylor et al., 2004) was used to measure heritage cultural identity exploration (six items, ω = 0.79–83) and resolution (four items, ω = 0.85–86), while in Germany, the Ethnic Identity Scale - Brief (Douglass & Umaña-Taylor, 2015), with three items from both the exploration (ω = 0.84–89) and resolution (ω = 0.76–91) subscales of the Ethnic Identity Scale were used. Sample items are “I have attended events that have helped me learn more about my culture of origin (Italy)/cultural group (Germany)” (exploration) and “I have a clear sense of what my culture of origin (Italy)/ heritage culture (Germany) means to me” (resolution). The response scale ranged from 1 = No, that’s not true to 4 = Yes, that’s true.

#### Classroom cultural diversity climate

Three subscales of the Classroom Cultural Diversity Climate Scale (Schachner et al., 2021) were used in this study: heritage and intercultural learning (7 items; e.g., “In class, we learn about the heritage cultures of the students in my class”, ω_Italy_ = 0.84–0.86, ω_Germany_ = 0.83–0.88), critical consciousness (5 items; e.g., “In class, we talk about the fact that people from different backgrounds do not always have the same opportunities in Germany/Italy”, ω_Italy_ = 0.72–0.82, ω_Germany_ = 0.76–0.82), and equal treatment (5 items; e.g., “Students of some heritage cultures can get away with more in class than students of other heritage cultures” – reversed, ω_Italy_ = 0.71–0.85, ω_Germany_ = 0.71–0.79). The response scale ranged from 1 = No, that’s not true to 5 = Yes, that’s true.

### Analytic Plan

Analyses were conducted using version 4.1.0 (R Core Team, 2023), RStudio version 2023.3.1.44 (Posit team, 2023), and the brms package (Bürkner, 2021). Due to Covid-19, the data includes missing waves on classroom and individual level. Only complete cases with no missing values were included in the analyses. To test for the likelihood of our results being influenced by missing values, we compared the main study variables and demographics among adolescents who had vs. did not have missing data at mean level using independent t-tests, and logistic regressions. If there were differences on a demographic variable, this was included in the analyses as a control variable. Bayesian multivariate linear models were chosen to test our hypotheses, as they provide flexibility in specifying prior distributions for model parameters, which allows us to incorporate prior knowledge or beliefs about the data into the analysis. Moreover, these models can simultaneously analyze multiple outcome variables, allowing us to examine the complex relationships in question in a single framework. Lastly, Bayesian methods naturally provide estimates of uncertainty through posterior distributions, allowing us to quantify the uncertainty associated with model parameters and predictions, rather than merely categorizing associations as significant vs. non-significant. Due to slight differences in time points, sample ages and ethnic identity measures, analyses were run separately for the Italian and German subsamples. To test the hypotheses, first, multivariate linear models (see conceptual model, Fig. 1) were run in the larger Italian sample.

Priors for the Bayesian multivariate linear models were chosen based on theoretical considerations and anticipated relationships among the variables (Supplementary Online Resource 1 and 2). We utilized the student t distribution to define our priors, where the first argument represents the degrees of freedom, the second argument denotes the mean of the distribution, and the third argument signifies the scale or standard deviation of the distribution. For cases where we expected no difference, such as the effect of age on critical consciousness classroom climate at T2, we selected a mean that reflected no difference (0) while incorporating some level of uncertainty (0.5). Conversely, in instances where we were confident in expecting a relationship, such as predicting critical consciousness classroom climate at T2 based on T1, we set the mean accordingly (0.3) and expressed greater certainty by reducing the standard deviation (0.1). We chose associations around 0.3 as they are commonly observed in psychological research. Concretely this means that when considering a student’s t(3,3,0.1) distribution means that we expect, with 90% probability, the parameter value to fall within the interval [2.76, 3.24].

Then, different variations of this model were run and compared (see Model Fit). Normalcy and linearity of all model residuals were assessed with graphical representations. Models were further evaluated with leave-one-out cross-validation (LOO) and the Bayesian R^2^. Furthermore, using established practices (e.g., Lionetti et al., 2022), robustness checks were run using imputed data of the complete sample, including cases with missing values. Further, in the German sample the results from the Italian sample were used as informative priors, i.e., the Italian results were used as priors in the German model (Supplementary Online Resource 2).

## Results

### Descriptives

Means, SDs, measurement invariance and correlations among variables of interest are reported in Supplementary Online Resource 3–7. Tests for mean differences between adolescents in Italy and Germany on the main study variables suggest that exploration was higher in Italy than in Germany at all three time points, whereas the resolution was higher in Germany than in Italy at all three time points (Cohen’s d for all of them indicating small effects, approaching medium). As for the classroom cultural diversity climate, heritage and intercultural learning was higher (medium effect) in Germany than in Italy at all three time points, whereas critical consciousness socialization was higher in Germany only at T3 (small effect).

In the Italian subsample, 25% of participants had missing data, while complete data were available for *N* = 718 students (30.64% of immigrant descent, *M*_age_ = 15.06 years, *SD*_age_ = 0.65, 54.04% self-identifying as female). In the German subsample, 53% of participants had missing data, while complete data were available for *N* = 272 students (49.26% of immigrant descent, *M*_age_ = 12.76 years, *SD*_age_ = 0.78, 48.53% self-identifying as female). Evaluation of sample distributions with and without missing values showed minimal differences (smallest overlapping distribution area = 0.77%, see Online Resource 14). Across both samples, a higher share of students with missing data were of immigrant descent (63%) compared to students without missing data (49%). Also, in the Italian sample, students with missing data were slightly older (*M* = 15.32) than students without missing data (*M* = 15.06), therefore age was added as control variable in the Italian model (Graham, 2003). Moreover, a higher share of students with missing data were in the intervention condition (58%) compared to students without missing data (51%). In the German sample, students with missing data had slightly higher SES (*M* = 4.72) than students without missing data (*M* = 4.53), therefore SES was added as control variable in the German model. No differences in missingness by gender emerged in either sample. Differences between data with and without missing values on the main study variables are reported in Supplementary Online Resource 14.

### Model Fit and Evaluation

In both the Italian and German sample, a full model including all hypotheses and the control variables immigrant descent and age/SES were compared to a null model including no effects, a null model including random intercepts, a full model without age/SES, a full model without age/SES and immigrant descent, and a full model without stability coefficients (for a detailed overview of models, see Supplementary Online Resource 15 and 16). Then, coefficients were removed step-by-step from the most complex model (hypothesized model including immigrant descent and auxiliary variables age/SES) until LOO indicated the most likely model to describe the data was found. The hypothesized and final models for Italy and Germany can be seen in Table 1. A total of 218 (Italy) and 207 (Germany) models were estimated and compared. The final models for both countries do not explain more variance than the hypothesized full model, including or excluding immigrant descent or age/SES as controls (Bayesian R^2^, see Tables 2 and 3). While most of the variance is explained by stability coefficients, comparisons of LOO weights show that the final models are over 995 times more likely than the other models.

**Table 1:** Overview of the coefficients of the hypothesized model including immigrant descent, and the models evaluated as most likely for the Italian and German sample

	Hypothesized Model	Final Model Italy	Final Model Germany
Heritage and intercultural learning T2~	Heritage and intercultural learning T1+	Heritage and intercultural learning T1+	Heritage and intercultural learning T1+
	Intervention+		Intervention+
	Immigrant descent+	Immigrant descent+	
	(1 | class ID)	(1 | class ID)	(1 | class ID)
Critical consciousness T2~	Critical consciousness T1+	Critical consciousness T1+	Critical consciousness T1+
	Intervention+	Intervention+	intervention+
	Immigrant descent+		Immigrant descent+
	(1 | class ID)	(1 | class ID)	(1 | class ID)
Equal treatment T2~	Equal treatment T1+	Equal treatment T1+	Equal treatment T1+
	Intervention+		Intervention+
	Immigrant descent+		Immigrant descent+
	(1 | class ID)	(1 | class ID)	(1 | class ID)
Heritage cultural identity exploration T2~	Heritage cultural identity exploration T1+	Heritage cultural identity exploration T1+	Heritage cultural identity exploration T1+
	Critical consciousness T1+	Critical consciousness T1+	Critical consciousness T1+
	Equal treatment T1+		Equal treatment T1+
	Heritage and intercultural learning T1+		
	Intervention+	Intervention+	Intervention+
	Intervention * critical consciousness T1+	Intervention * critical consciousness T1+	
	Intervention * equal treatment T1+		Intervention * equal treatment T1+
	Intervention *heritage and intercultural learning T1+		
	Immigrant descent+	Immigrant descent+	Immigrant descent+
	(1 | class ID)	(1 | class ID)	(1 | class ID)
Heritage cultural identity resolution T3~	Heritage cultural identity resolution T1+	Heritage cultural identity resolution T1+	Heritage cultural identity resolution T1+
	Heritage cultural identity exploration T2+	Heritage cultural identity exploration T2+	
	Critical consciousness T2+	Critical consciousness T2+	
	Equal treatment T2+		Equal treatment T2+
	Heritage and intercultural learning T2+	Heritage and intercultural learning T2+	
	Intervention+	Intervention+	
	Intervention * critical consciousness T2+		
	Intervention * equal treatment T2+		
	Intervention *heritage and intercultural learning T2+	Intervention* heritage & intercultural learning T2+	
	Immigrant descent+	Immigrant descent+	Immigrant descent+
	(1 | class ID)	(1 | class ID)	(1 | class ID)

**Table 2:** Comparison of a selection of models for the Italian sample

	Bayesian R^2^ per dependent variable					L00 model weight
	Heritage and intercultural learning T2	Critical consciousness T2	Equal treatment T2	Heritage cultural identity exploration T2	Heritage cultural identity resolution T3	
Null model	0.00	0.00	0.00	0.00	0.00	0.000
Null model including random intercepts	0.01	0.03	0.12	0.02	0.02	0.000
Hypothesized model without stability coefficients	0.02	0.03	0.12	0.04	0.43	0.000
Hypothesized model	0.27	0.24	0.28	0.38	0.44	0.000
Hypothesized model including immigrant descent	0.27	0.24	0.28	0.38	0.44	0.001
Hypothesized model including immigrant descent and age	0.28	0.24	0.28	0.38	0.44	0.000
Final model	0.27	0.24	0.28	0.38	0.43	0.998

Bayesian R^2^ shows the proportion of variance explained by the defined model in each level of the multilevel model. Loo (leave-one-out cross validation) model weights show likelihood of the model compared to other models

**Table 3:** Comparison of a selection of models for the German sample

	Bayesian R^2^ per dependent variable					L00 model weight
	Heritage and intercultural learning T2	Critical consciousness T2	Equal treatment T2	Heritage cultural identity exploration T2	Heritage cultural identity resolution T3	
Null model	0.00	0.00	0.00	0.00	0.00	0.000
Null model including random intercepts	0.09	0.06	0.04	0.05	0.09	0.000
Hypothesized model without stability coefficients	0.09	0.06	0.05	0.09	0.27	0.000
Hypothesized model	0.24	0.27	0.16	0.31	0.36	0.000
Hypothesized model including immigrant descent	0.25	0.28	0.16	0.34	0.39	0.005
Hypothesized model including immigrant descent and FASII	0.25	0.28	0.16	0.34	0.39	0.000
Final model	0.24	0.27	0.16	0.34	0.33	0.995

Bayesian R^2^ shows the proportion of variance explained by the defined model in each level of the multilevel model. Loo (leave-one-out cross validation) model weights show likelihood of the model compared to other models

#### Evaluation of distributions

Once the final model was identified, posterior distributions of model parameters were interpreted. Each posterior was summarized by its mean value and 95%-CI. Additionally, we evaluated effects considering the region of practical equivalence (ROPE; Kruschke, 2014), which defines values that are equivalent to the null effect. The ROPE was set from −0.1 to +0.1 for all model parameters. The lower the percentage of overlap between the ROPE and the highest posterior density interval (HPDI), the stronger the support for the investigated effect. To evaluate differences between our hypothesized/prior distributions and posterior distributions, we compared prior and posterior density intervals (see Supplementary Online Resource 17 and 18). In other words, after choosing the best model for the data, the range of possible values for each parameter was analyzed to determine whether these values were practically the same as no effect, and the beliefs about the relationships between variables before and after analyzing the data were compared.

#### Robustness check – imputation

To check robustness, data were imputed using the R mice package (van Buuren & Groothuis-Oudshoorn, 2011) and differences from the model run with list-wise deletion were explored. As results were very similar for imputed and non-imputed data, we report only the latter. Results from the imputed data can be found in Supplementary Online Resource 19 and 20. Additionally, a robustness test was conducted with a model including additional covariates, controlling for immigrant generation, percentage of students of immigrant descent per class, school type, covid-19 pandemic and gender. However, results showed that the model without the additional covariates better fit the data, and therefore only the final model is reported. Results from both the robustness test with additional covariates can be found in Supplementary Online Resource 21 and 22.

### Final Model Results - Italy

#### Research Question 1: Main effects of Identity Project and climate on identity

**Fig. 2:**
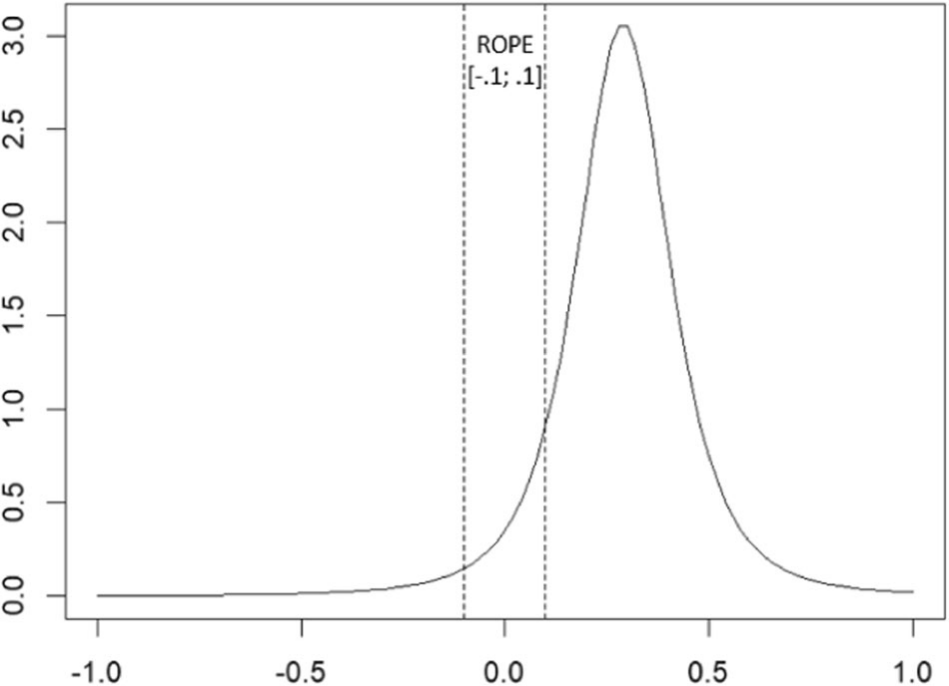
Distribution of the effect of intervention on heritage cultural identity exploration at T2 (degrees of freedom = 3.00, mean of the distribution = 0.29, standard deviation of the distribution = 0.12). The x-axis represents the range of values in our data, describing the association between the intervention and heritage cultural identity exploration at T2. The y-axis describes the probability density values (density) associated with each of the corresponding x-values within that range. A higher density at a particular value on the x-axis indicates that the corresponding value or range of values is more likely to occur in the underlying probability distribution. All values inside the ROPE are considered null effects, and all values outside the ROPE are considered effects. The lower the percentage of overlap between the ROPE and the highest posterior density interval, the stronger the support for the investigated effect. In this case, there is a 95% probability of an effect, as 95% of the density is being above the ROPE [−0.1; 0.1]

The intervention group predicted an increase of heritage cultural identity exploration at T2 (*β* = 0.28 [0.06, 0.52]), with a 95% probability of an effect being above the ROPE^2^ (Fig. 2). For the classroom cultural diversity climate, neither heritage culture and intercultural learning nor equal treatment at T1 could explain variance of T2 exploration in the final model, while critical consciousness at T1 (*β* = 0.05 [−0.01., 0.12]) was positively associated with T2 exploration, with an 8% probability of the effect lying above the ROPE. Additionally, heritage cultural identity exploration at T1 (*β* = 0.52 [0.47, 0.57], HPDI above the ROPE = about 99%) and being a student of immigrant descent (*β* = 0.10 [0.03, 0.17], HPDI above the ROPE = 56%) were associated with increased heritage cultural identity exploration at T2.

The intervention group also predicted heritage cultural identity resolution at T3 as expected (*β* = 0.44 [0.12, 0.75]), with a probability of 98% of being above the ROPE. The indirect effects of the intervention on resolution at T3 via exploration at T2 was *β* = 0.11 [0.02, 0.18] (HPDI above ROPE = 53%). A more positive classroom cultural diversity climate as measured with heritage and intercultural learning at T2 (*β* = 0.17 [0.01, 0.34]) also increased heritage cultural identity resolution at T3, with an 80% probability of being above the ROPE. However, against our expectations, critical consciousness at T2 was negatively associated with resolution at T3 (*β* = −0.06 [−0.20, 0.07], HPDI below the ROPE = 29%, HPDI above ROPE = 1%). There was no indirect effect of critical consciousness at T1 on resolution at T3 via exploration at T2, with *β* = 0.02 [−0.00, 0.04] (HPDI outside the ROPE = 0%).

Additionally, heritage cultural identity exploration at T2 (*β* = 0.37 [0.24, 0.50]) and heritage cultural identity resolution at T1 (*β* = 0.40 [0.34, 0.46]) were associated with an increased heritage cultural identity resolution at T3 (both HPDI above the ROPE = about 99%). Being a student of immigrant descent (*β* = 0.08 [−0.01, 0.17]) was related to an increase in resolution at T3 (HPDI above the ROPE = 32%).

#### Research Question 2: Main effect of Identity Project on climate

The intervention group helped explain variance in only one of the three aspects of cultural diversity climate: critical consciousness (*β* = 0.07 [−0.03, 0.17]), with a 25% probability of a positive effect of intervention. Moreover, climate at T1 was a large predictor of climate at T2 (heritage and intercultural learning *β* = 0.41 [0.36, 0.46]; critical consciousness socialization *β* = 0.48 [0.42, 0.54]; equal treatment *β* = 0.51 [0.44, 0.59]; all three HPDI above the ROPE = about 99%). Students of immigrant descent perceived less heritage culture and intercultural learning at T2 than students of non-immigrant descent (*β* = −0.04 [−0.11, 0.02], HPDI below the ROPE = 5%).

#### Research Question 3: Climate moderating Identity Project effects on identity

**Fig. 3:**
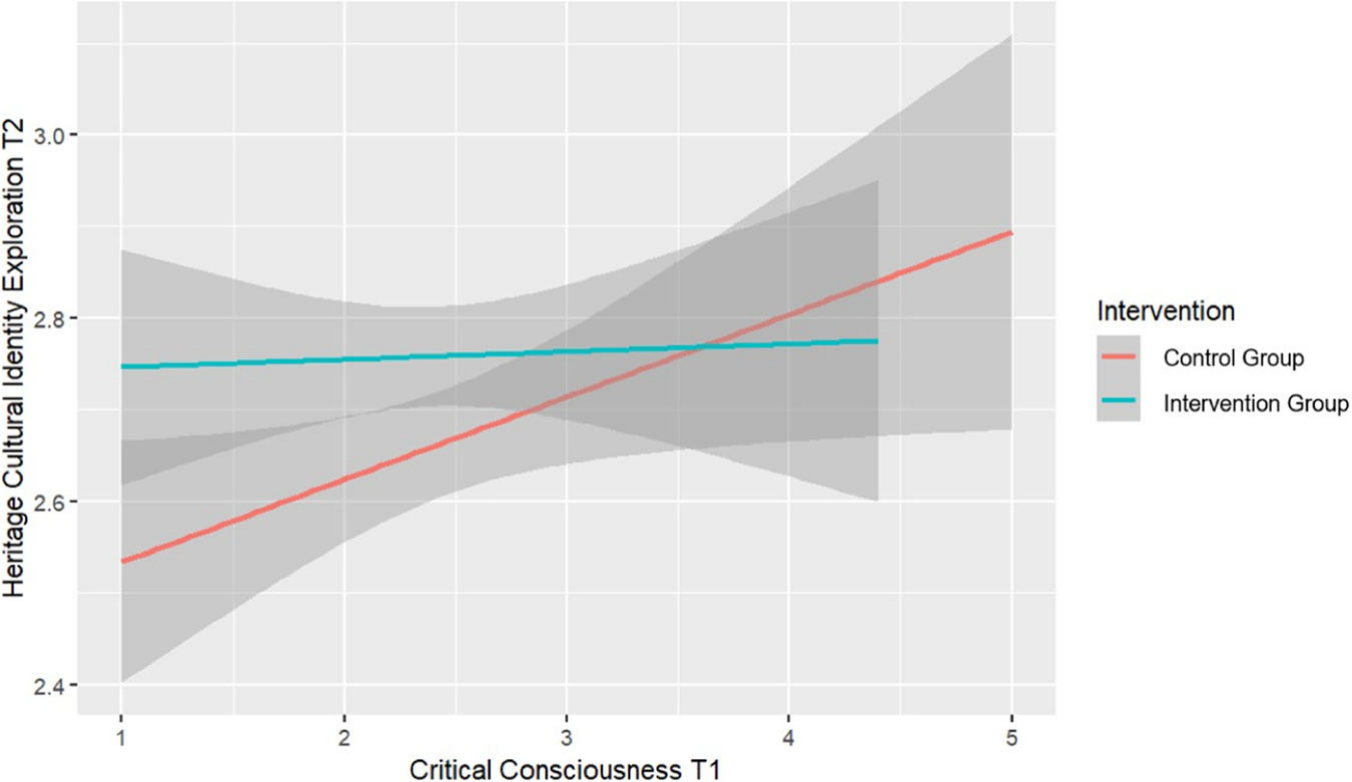
Interaction Intervention and Critical Consciousness T1 on Heritage Cultural Identity Exploration at T2 - Italy

The intervention effect on heritage cultural identity exploration at T2 interacted with critical consciousness socialization at T1 (*β* = −0.08 [−0.17, 0.01], HPDI below the ROPE = 33%). As seen in Fig. 3, critical consciousness socialization at T1 predicted increased exploration for students in the control group, but not in the intervention group.

**Fig. 4:**
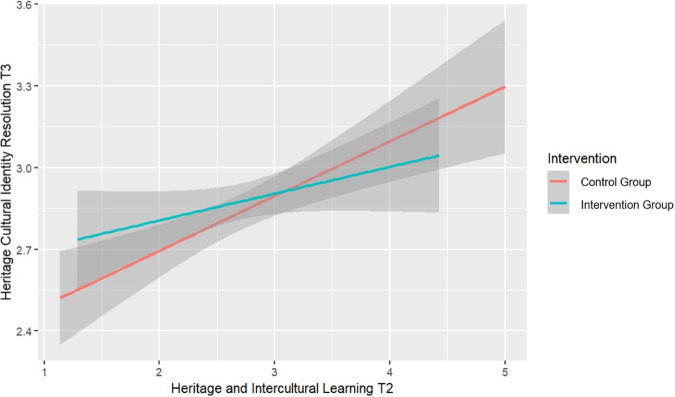
Interaction Intervention and heritage and Intercultural Learning at T2 on Heritage Cultural Identity Resolution at T3 - Italy

Moreover, the intervention effect on heritage cultural identity resolution at T3 interacted with heritage culture and intercultural learning at T2 (*β* = −0.15 [−0.27, −0.04], HPDI below the ROPE = 82%). As seen in Fig. 4, heritage culture and intercultural learning at T2 predicted resolution more strongly for students in the control group, than in the intervention group.

### Final Model Results - Germany

#### Research Question 1: Main effects of Identity Project and climate on identity

The intervention group predicted an increase of heritage cultural identity exploration at T2 (*β* = 0.30 [0.10, 0.51]), with a probability of 98% of an effect above the ROPE. Classroom climate at T1 measured as critical consciousness was positively associated with exploration (*β* = 0.04 [−0.03, 0.10], HPDI above the ROPE = 3%), and equal treatment was negatively associated with exploration (*β* = −0.03 [−0.12, 0.04], HPDI below the ROPE = 6%). Heritage culture and intercultural learning at T1 did not help explain heritage cultural identity exploration at T2. However, heritage cultural identity exploration at T1 (*β* = 0.53 [0.47, 0.59], HPDI above the ROPE = about 99%) and being a student of immigrant descent (*β* = 0.31 [0.15, 0.47], HPDI above the ROPE = about 99%) were associated with an increased heritage cultural identity exploration at T2.

The intervention, heritage and intercultural learning at T2, and critical consciousness at T2 did not help explain heritage cultural identity resolution at T3. Equal treatment at T2 is mostly positively associated resolution at T3 (*β* = 0.02 [−0.11, 0.17], HPDI below the ROPE = 3%, HPDI above the ROPE = 12%). Additionally, heritage cultural identity resolution at T1 (*β* = 0.43 [0.37, 0.51], HPDI above the ROPE = about 99%) and being a student of immigrant descent (*β* = 0.36 [0.22, 0.52], HPDI above the ROPE = about 99%) were positively associated with an increased heritage cultural identity resolution at T3.

#### Research Questions 2 and 3: Main effect of Identity Project on climate and climate moderating Identity Project effects on identity

The intervention group predicted a more positive cultural diversity climate: heritage culture and intercultural learning (*β* = 0.05 [−0.04, 0.15], HPDI above the ROPE = 12%), critical consciousness (*β* = 0.06 [−0.06, 0.17], HPDI below the ROPE = 1%, HPDI above the ROPE = 24%), and equal treatment (*β* = −0.06 [−0.17, 0.07], HPDI below the ROPE = 23%, HPDI above the ROPE = 1%). Climate at T1 was a large predictor of climate at T2 (heritage and intercultural learning *β* = 0.39 [0.33, 0.43]; critical consciousness *β* = 0.46 [0.40, 0.51]; equal treatment *β* = 0.34 [0.22, 0.46]; all three HPDI above the ROPE = about 99%), and being a student of immigrant descent was negatively related to the perception of the cultural diversity climate at T2 (critical consciousness *β* = −0.08 [−0.26, 0.10], HPDI below the ROPE = 42%, HPDI above the ROPE = 2%; equal treatment *β* = −0.17 [−0.38, 0.03], HPDI below the ROPE = 76%). There was no moderating effect of intervention and climate on exploration at T2 or resolution at T3.

## Discussion

There are still relatively few studies of ethnic-racial identity development in Europe compared to the United States (Erentaitė et al., 2018). At the same time, previous research has not considered the immediate classroom context and how it may interact with the intervention or be changed as a result of the intervention, nor tested efficacy in a younger age group of early adolescents. Our study addresses these gaps, investigating the dynamic interplay of the Identity Project as a curriculum-based intervention with the classroom cultural diversity climate in relation to adolescents’ heritage cultural identity development. By testing associations amongst mid-adolescents in Italy, and then amongst early adolescents in Germany, it contributes to a better understanding of how social contexts affect and are affected by a curriculum-based intervention like the Identity Project.

### Classroom Cultural Diversity Climate and Intervention Facilitating Identity Development

Mostly in line with previous US studies (Umaña-Taylor, Douglass, et al., 2018; Umaña-Taylor, Kornienko, et al., 2018), adolescents in the intervention group (vs. control group) showed increased heritage cultural identity exploration at T2 in Italy and Germany, while an intervention effect on resolution at T3, directly and indirectly through exploration at T2, was only confirmed in Italy (Hypothesis 1a). It should be noted though, that our findings are more robust than those of the original studies around the Identity Project as we also control for T1 outcome measures. One reason for the absence of an effect on T3 resolution in Germany could be that for the younger sample, it may take a longer period of exploration in the course of adolescence before this is connected to an increase in resolution. Interestingly, resolution was higher in Germany than in Italy at all three time points, whereas exploration was lower. Together, this resembles a pattern of foreclosure (Marcia, 1980; Phinney, 1989), where commitment or resolution is achieved without prior exploration. At the same time, newer research suggests that the relation between exploration and commitment may not be as linear as previously proposed, but is rather a more reciprocal, parallel process (Crocetti, 2017). According to these newer conceptualizations of identity development, youth may already be committed to certain identities when entering adolescence (showing higher levels of resolution as we observed them in Germany), but first engage in a formation cycle, where existing commitments are reconsidered in light of additional possible identities, before engaging in a maintenance cycle where commitment is linked to in-depth exploration.

Nevertheless, given the effects of the intervention on exploration in both countries, it is encouraging to see that the engaging with ethnic, racial and cultural diversity as part of the curriculum can already promote ethnic-racial or heritage culture identity development in younger adolescents. As noted before, ethnic-racial identity is connected with a wide range of positive development outcomes, including school adjustment (Umaña-Taylor & Rivas-Drake, 2021), and there is also evidence from the United States for effects of the Identity Project on these outcomes (Umaña-Taylor, Kornienko, et al., 2018). Intervening earlier allows for effects to unfold in time to influence the achievement of important milestones, such as school completion and better final grades, thereby reducing the detrimental effects of the opportunity gap that systematically disadvantages children and youth of immigrant descent.

The broader classroom context captured by the cultural diversity climate also mattered for heritage cultural identity development (partly supporting Hypothesis 1b). Specifically, adolescents in Italy and Germany who perceived a stronger classroom climate of critical consciousness before the intervention showed more heritage culture identity exploration at T2. Concerning effects of the classroom cultural diversity climate at T2, a positive effect of heritage and intercultural learning and an unexpected negative effect of critical consciousness climate on T3 resolution was observed in Italy, but not in Germany. One reason for the absence of effects on resolution in Germany could be the younger sample, as also discussed in relation to the absence of an Identity Project effect on resolution above. The unexpected negative effect of critical consciousness climate on resolution in Italy could be due to the overall context and climate in Italian schools being perceived as less supportive towards the needs of youth of immigrant descent, pressuring them to assimilate. This less supportive climate is also reflected in a lower score on the Multiculturalism Policy Index (2021) compared to Germany, and the recent election of a right-wing government. In this less supportive climate, reflecting and discussing societal inequities at school may not be coupled with efficacy or action to confront these inequities. Indeed, a recent analysis of individual critical consciousness profiles of adolescents shows that if reflection is not coupled with action, this can promote negative socio-emotional outcomes (Schwarzenthal et al., 2023). Finally, equal treatment showed no associations with heritage culture identity exploration and resolution in Italy, but negative associations in Germany. This corresponds with previous research, showing either no relation (Juang et al., 2023; Schachner et al., 2016) or a negative relation (Aral et al., 2023) of equal treatment climate with heritage culture identity. One explanation is that equal treatment sometimes reflects color-evasion, consciously neglecting and ignoring diversity. Another explanation could be that, as in other studies, we measured equal treatment with reverse-items, indicating the absence of inequality, whereby inequality was found to be associated with stronger heritage culture identities (Fleischmann et al., 2019), in line with rejection-identification hypothesis (Branscombe et al., 1999).

### Engaging with Diversity in the Curriculum as a Means to Change the Classroom Cultural Diversity Climate

Next, we wanted to see if engaging with cultural diversity in the curriculum would also promote a better classroom cultural diversity climate (Hypothesis 2). Indeed, adolescents who went through the intervention perceived a stronger climate of critical consciousness at T2 in Italy and Germany, while effects on heritage culture and intercultural learning and on equal treatment were only observed in Germany. Yet, intervention effects on classroom cultural diversity climate were generally small. Concerning the different pattern across countries, it is possible that in Italy and with older adolescents and in higher grades, the classroom diversity climate was already more established prior to the intervention, so that there was less room for the intervention to still change it. One exception is critical consciousness climate, which may require a higher level of abstraction and may therefore become more relevant in later adolescence, allowing it still to be changed by the intervention also in Italy. Another reason why we only found intervention effects on the classroom cultural diversity climate in Germany may be that the intervention and the broader school context were more disconnected, and the overall context less supportive in Italy. Besides a generally less diversity-friendly climate as mentioned above, findings from the Program for International Student Assessment showed that teacher-student relationships were less positive and below OECD average in Italy compared to Germany (OECD, 2012). This corresponds with anecdotal evidence from student feedback rounds after the completion of the Identity Project in Italy, that they did not want their own teachers to deliver the intervention. The intervention may therefore have fallen on less fertile grounds in Italy in terms of the possibility to improve the classroom cultural diversity climate (Walton & Yeager, 2020).

### Can a Positive Classroom Cultural Diversity Climate Boost Intervention Effects on Identity?

Finally, we found very little support for interactive effects between the Identity Project and the classroom cultural diversity climate on heritage culture identity development. Based on previous research on parental ethnic-racial socialization boosting intervention effects (Sladek et al., 2021), we expected that the Identity Project would be even more efficacious in a more positive classroom cultural diversity climate (Hypothesis 3). Instead, we found evidence of compensatory effects, where climate effects are stronger when they are not coupled with the intervention. Specifically, in Italy a stronger critical consciousness climate at T1 was associated with increased exploration at T2 only for students in the control group, but not in the intervention group, while heritage and intercultural learning at T2 predicted resolution at T3 more strongly for students in the control group, than in the intervention group. Heritage and intercultural learning was also lower at all three time points in Italy compared to Germany, whereas perceptions of the other two dimensions of the classroom cultural diversity climate were more similar across countries. Thus, if engaging with diverse heritage cultures in class is less normative, as we observe it in Italian schools compared to German schools, it may have a greater effect when it does occur, but at the same time, when coupled with the Identity Project, the unique contribution of the climate beyond the intervention is smaller.

### Limitations and Future Directions

Employing rigorous, Bayesian analyses and testing our hypotheses in two samples in Italy and Germany are clear strengths of our study. Yet, especially in the German subsample we had a relatively high proportion of missing values, which was mostly due to students missing entire waves of data collection because of a high Covid- and quarantine-related absence rate. There was also a Covid-related school closure in the 2019/20 cohort in Germany, meaning that the 3rd wave of data could not be collected. We tried to accommodate this as much as possible by relying only on complete data and at the same time conducting various robustness checks. Nevertheless, it would underline the validity of our findings to confirm them also under non-Covid conditions. Besides the high missingness rate, this extra-ordinary time may also have had implications for some of our core constructs, such as by limiting possibilities for cultural identity exploration (Ceccon, Schachner, Umaña-Taylor, et al., 2023) and possibly making it harder to have a clear sense of the cultural diversity climate in schools when school experiences were so interrupted. This could also be one reason why measurement invariance across time was not achieved for some of our core constructs.

Replicating original efficacy studies from the United States (Umaña-Taylor, Douglass, et al., 2018; Umaña-Taylor, Kornienko, et al., 2018), we also tested a cascading model where the Identity Project first increases cultural identity exploration, which in turn is associated with increased resolution. Yet, drawing on newer research on identity development in adolescence more broadly (Crocetti, 2017), future studies testing effects of the Identity Project and classroom cultural diversity climate should also test for parallel and reciprocal effects on exploration and resolution, and also include reconsideration of commitment as an additional process, which may be particularly relevant in early adolescence. Future research could also include content in addition to process dimensions of identity (Umaña-Taylor et al., 2014), such as centrality, public and private regard.

The Identity Project was originally developed as a universal intervention (Umaña-Taylor, Kornienko, et al., 2018). However, there is also some evidence from the US that effects may vary between heritage groups and belonging to a more privileged or more marginalized group (Sladek et al., 2021). Unfortunately, the size and high level of diversity of the sample in our study did not allow for subgroup analyses. However, it would be interesting for future research to investigate this, possibly also employing continuous moderators getting directly at marginalization experiences, such as experiences of ethnic-racial discrimination or foreigner objectification.

Finally, as in most previous studies around the Identity Project, the curriculum was implemented by researchers and trained facilitators external to the schools. Yet, recent research shows that the intervention can be even more effective in terms of promoting ethnic-racial identity development if implemented by teachers regularly teaching in the class (Umaña-Taylor et al., 2023). Especially concerning intervention effects on the classroom cultural diversity climate, it seems likely that these would be larger if the program was delivered by teachers who then also continue to teach the class. In fact, teachers themselves might change as a result of the intervention in terms of their culturally responsive teaching skills. If implemented in this way, the Identity Project may also be an avenue for whole-school development, tackling educational inequality at the structural level. Yet, if teachers are to implement the program, they have to be carefully trained to deliver the program in the intended way and be sensitive to their students’ identity-related needs and vulnerabilities.

## Conclusion

While both the classroom cultural diversity climate and curriculum-based interventions can promote cultural identity development, they have not been studied together. Drawing on theories of ethnic-racial identity development, the current study aimed to understand the dynamic interplay of a curriculum-based intervention (the Identity Project) with the classroom cultural diversity climate (heritage culture and intercultural learning, critical consciousness socialization and equal treatment) on cultural identity exploration and resolution. We first tested associations amongst Italian mid-adolescents and then with early adolescents in Germany. The Identity Project as well as a stronger critical consciousness climate in the classroom before the intervention promoted cultural identity exploration at post-test in both countries. However, effects of the intervention and facets of the diversity climate on resolution were only observed in the older sample in Italy. There is also some evidence that the Identity Project can alter the classroom cultural diversity climate, but this was mostly observed in the younger German sample, where the climate may still be more malleable. While we expected that a positive cultural diversity climate would boost intervention effects, we saw a compensatory effect in Italy. Especially in an overall less diversity-friendly context like in Italy, the intervention may therefore be a way to provide a protective space in the classroom. Taken together, both aspects of the broader classroom cultural diversity climate and providing opportunities to engage with cultural diversity and students’ own cultural backgrounds in class can promote cultural identity development in adolescence. In addition, implementing a curriculum like the Identity Project in schools may also contribute to a better cultural diversity climate, especially if the project is implemented earlier in adolescents’ secondary school trajectory. Thus, it seems promising to systematically build in opportunities to engage with students’ diverse heritage cultures and identities when developing new curricula, as well as to train teachers to implement such curricula.

## Supplementary Information

Supplementary Information

## Footnotes

1 We use “cultural identity” when discussing studies in Europe and “ethnic-racial identity” when referring to studies in the United States, taking into account socio-cultural variations in the understanding of ethnicity, race, and culture. Cultural identity can be based on national (e.g. German), regional (e.g. Bavarian), ethnic (e.g. Sorbian), religious (e.g. Muslim) affiliation(s) most relevant to the self-concept of individuals. Some studies in Europe drawing on an acculturation framework also distinguish between heritage and national components of cultural identity, with the former referring to the familial background of a person and the latter to the society they live in. (for a review of this conceptual debate and practices in Europe, see Juang et al., 2022).

2 The HPDI outside the ROPE represents values indicating associations between the two variables in question that are not considered null effects. Values inside the ROPE are considered null effects. HPDIs above the ROPE are values larger than 0.1, and HPDIs below the ROPE are values smaller than −0.1. The HPDI outside the ROPE is reported one-sidedly if the HPDI on one side of the ROPE equals 0.00, meaning there are no values either above or below the ROPE, indicating that based on the observed data, there is no probability of a positive or negative relationship between the two variables in question.
