# Supplementary material for: Adolescent Cultural Identity Development in Context: The Dynamic Interplay of the Identity Project With Classroom Cultural Diversity Climate in Italy and Germany
Source: J Youth Adolesc. 2024 Jun 28;53(11):2480–98. doi: 10.1007/s10964-024-02031-5 (PMC11467110; doi:10.1007/s10964-024-02031-5)
Supplement: Supplementary file 1 — Supplementary Information [file 10964_2024_2031_MOESM1_ESM.docx]

**Supplementary Information**

**Adolescent cultural identity development in context: The dynamic interplay of the Identity Project with classroom cultural diversity climate in Italy and Germany**

[Online Resource 1. *Priors for parameters in the Italian models.* 2](#_Toc164248873)

[Online Resource 2. *Priors for parameters in the German models.* 4](#_Toc164248874)

[Online Resource 3. *Descriptives of main study variables for Italy and Germany, total sample.* 6](#_Toc164248875)

[Online Resource 4. *Differences of main study variables per time point Italy.* 8](#_Toc164248876)

[Online Resource 5. *Differences of main study variables per time point Germany.* 9](#_Toc164248877)

[Online Resource 6. *Descriptives of main study variables for Italy, comparing students of immigrant descent and non-immigrant descent..* 10](#_Toc164248878)

[Online Resource 7. *Descriptives of main study variables for Germany, comparing students of immigrant descent and non-immigrant descent..* 12](#_Toc164248879)

[Online Resource 8. *Measurement invariance Italian sample, comparing students of immigrant descent and non-immigrant descent.* 14](#_Toc164248880)

[Online Resource 9. *Measurement invariance German sample, comparing students of immigrant descent and non-immigrant descent.* 22](#_Toc164248881)

[Online Resource 10. *Longitudinal measurement invariance Italian sample.* 30](#_Toc164248882)

[Online Resource 11. *Longitudinal measurement invariance German sample.* 33](#_Toc164248883)

[Online Resource 12. *Correlations main study variables and controls – Italy.* 36](#_Toc164248884)

[Online Resource 13. *Correlations main study variables and controls – Germany.* 37](#_Toc164248885)

[Online Resource 14. *Significant (α = 0.05) differences between students with and without missing data, Italy and Germany.* 38](#_Toc164248886)

[Online Resource 15. *Model comparisons – Italian sample.* 39](#_Toc164248887)

[Online Resource 16. *Model comparisons – German sample.* 42](#_Toc164248888)

[Online Resource 17. *Probability of effect prior and post – final model Italy.* 45](#_Toc164248889)

[Online Resource 18. *Probability of effect prior and post – final model Germany.* 46](#_Toc164248890)

[Online Resource 19. *Robustness check comparing list-wise deletion and imputed data - Italian sample.* 47](#_Toc164248891)

[Online Resource 20*. Robustness check comparing list-wise deletion and imputed data - German sample.* 49](#_Toc164248892)

[Online Resource 21. *Robustness check with additional covariates – Italian sample.* 51](#_Toc164248893)

[Online Resource 22. *Robustness check with additional covariates - German sample.* 55](#_Toc164248894)

# Online Resource 1. *Priors for parameters in the Italian models.*

| **Dependent variable** | **Predictor** | **Prior** |
| --- | --- | --- |
| Critical consciousness T2 | Critical consciousness T1 | student_t(3, .3, .1) |
| Critical consciousness T2 | Intervention | student_t(3, .3, .5) |
| Critical consciousness T2 | Immigrant descent | student_t(3, 0, .5) |
| Critical consciousness T2 | Age | student_t(3, 0, .5) |
| Equal treatment T2 | Equal treatment T1 | student_t(3, .3, .1) |
| Equal treatment T2 | Intervention | student_t(3, 0, .5) |
| Equal treatment T2 | Immigrant descent | student_t(3, 0, .5) |
| Equal treatment T2 | Age | student_t(3, 0, .5) |
| Heritage and intercultural learning T2 | Heritage and intercultural learning T1 | student_t(3, .3, .1) |
| Heritage and intercultural learning T2 | Intervention | student_t(3, .3, .5) |
| Heritage and intercultural learning T2 | Immigrant descent | student_t(3, 0, .5) |
| Heritage and intercultural learning T2 | Age | student_t(3, 0, .5) |
| Heritage cultural identity exploration T2 | Critical consciousness T1 | student_t(3, .3, .5) |
| Heritage cultural identity exploration T2 | Equal treatment T1 | student_t(3, 0, .5) |
| Heritage cultural identity exploration T2 | Heritage and intercultural learning T1 | student_t(3, .3, .5) |
| Heritage cultural identity exploration T2 | Heritage cultural identity exploration T1 | student_t(3, .3, .1) |
| Heritage cultural identity exploration T2 | Intervention | student_t(3, .3, .5) |
| Heritage cultural identity exploration T2 | Immigrant descent | student_t(3, .3, .1) |
| Heritage cultural identity exploration T2 | Age | student_t(3, 0, .5) |
| Heritage cultural identity exploration T2 | Intervention * critical consciousness T1 | student_t(3, .3, .5) |
| Heritage cultural identity exploration T2 | Intervention * equal treatment T1 | student_t(3, 0, .5) |
| Heritage cultural identity exploration T2 | Intervention * heritage and intercultural learning T1 | student_t(3, .3, .5) |
| Heritage cultural identity resolution T3 | Critical consciousness T2 | student_t(3, .3, .5) |
| Heritage cultural identity resolution T3 | Equal treatment T2 | student_t(3, 0, .5) |
| Heritage cultural identity resolution T3 | Heritage and intercultural learning T2 | student_t(3, .3, .5) |
| Heritage cultural identity resolution T3 | Heritage cultural identity exploration T2 | student_t(3, .3, .5) |
| Heritage cultural identity resolution T3 | Intervention | student_t(3, .3, .5) |
| Heritage cultural identity resolution T3 | Immigrant descent | student_t(3, .3, .1) |
| Heritage cultural identity resolution T3 | Age | student_t(3, 0, .5) |
| Heritage cultural identity resolution T3 | Intervention * critical consciousness T2 | student_t(3, .3, .5) |
| Heritage cultural identity resolution T3 | Intervention * equal treatment T2 | student_t(3, 0, .5) |
| Heritage cultural identity resolution T3 | Intervention * heritage and intercultural learning T2 | student_t(3, .3, .5) |
| Heritage cultural identity resolution T3 | Heritage cultural identity resolution T1 | student_t(3, .3, .1) |

*Note.* Priors for the Italian model parameters were decided based on theoretical knowledge and anticipated relationships of the variables. Priors are defined using the student t distribution, where the first argument is the degrees of freedom, the second argument is the mean of the distribution, and the third argument is the scale or standard deviation of the distribution.

# Online Resource 2. *Priors for parameters in the German models.*

| **Dependent variable** | **Predictor** | **Prior** |
| --- | --- | --- |
| Critical consciousness T2 | Critical consciousness T1 | student_t(3, .48, .03) |
| Critical consciousness T2 | Intervention | student_t(3, .09, .06) |
| Critical consciousness T2 | Immigrant descent | student_t(3, -.07, .05) |
| Critical consciousness T2 | FASII | student_t(3, 0, .50) |
| Equal treatment T2 | Equal treatment T1 | student_t(3, .52, .04) |
| Equal treatment T2 | Intervention | student_t(3, -.08, .06) |
| Equal treatment T2 | Immigrant descent | student_t(3, .02, .05) |
| Equal treatment T2 | FASII | student_t(3, 0, .50) |
| Heritage and intercultural learning T2 | Heritage and intercultural learning T1 | student_t(3, .41, .02) |
| Heritage and intercultural learning T2 | Intervention | student_t(3, .03, .04) |
| Heritage and intercultural learning T2 | Immigrant descent | student_t(3, -.07, .04) |
| Heritage and intercultural learning T2 | FASII | student_t(3, 0, .50) |
| Heritage cultural identity exploration T2 | Critical consciousness T1 | student_t(3, .04, .04) |
| Heritage cultural identity exploration T2 | Equal treatment T1 | student_t(3, .00, .04) |
| Heritage cultural identity exploration T2 | Heritage and intercultural learning T1 | student_t(3, .02, .04) |
| Heritage cultural identity exploration T2 | Heritage cultural identity exploration T1 | student_t(3, .52, .03) |
| Heritage cultural identity exploration T2 | Intervention | student_t(3, .45, .25) |
| Heritage cultural identity exploration T2 | Immigrant descent | student_t(3, .10, .04) |
| Heritage cultural identity exploration T2 | FASII | student_t(3, 0, .50) |
| Heritage cultural identity exploration T2 | Intervention * critical consciousness T1 | student_t(3, -.09, .06) |
| Heritage cultural identity exploration T2 | Intervention * equal treatment T1 | student_t(3, -.04, .05) |
| Heritage cultural identity exploration T2 | Intervention * heritage and intercultural learning T1 | student_t(3, .01, .06) |
| Heritage cultural identity resolution T3 | Critical consciousness T2 | student_t(3, -.06, .08) |
| Heritage cultural identity resolution T3 | Equal treatment T2 | student_t(3, .01, .06) |
| Heritage cultural identity resolution T3 | Heritage and intercultural learning T2 | student_t(3, .19, .09) |
| Heritage cultural identity resolution T3 | Heritage cultural identity exploration T2 | student_t(3, .37, .07) |
| Heritage cultural identity resolution T3 | Intervention | student_t(3, .15, .27) |
| Heritage cultural identity resolution T3 | Immigrant descent | student_t(3, .08, .04) |
| Heritage cultural identity resolution T3 | FASII | student_t(3, 0, .50) |
| Heritage cultural identity resolution T3 | Intervention * critical consciousness T2 | student_t(3, .05, .07) |
| Heritage cultural identity resolution T3 | Intervention * equal treatment T2 | student_t(3, .07, .05) |
| Heritage cultural identity resolution T3 | Intervention * heritage and intercultural learning T2 | student_t(3, -.19, .08) |
| Heritage cultural identity resolution T3 | Heritage cultural identity resolution T1 | student_t(3, .40, .03) |

*Note.* Priors for the German model parameters were based on posteriors of the Italian model. Priors are defined using the student t distribution, where the first argument is the degrees of freedom, the second argument is the mean of the distribution, and the third argument is the scale or standard deviation of the distribution.

# Online Resource 3. *Descriptives and mean differences of main study variables for Italy and Germany, total sample.*

|  |  | **Italy** | | | |  | | **Germany** | | | | |  | | **Cohen’s *d***  **[95% - CI]** | |
| --- | --- | --- | --- | --- | --- | --- | --- | --- | --- | --- | --- | --- | --- | --- | --- | --- |
|  |  | | ***N*** | ***M* (*SD*)** | **Range** | | **ω** | | ***N*** | ***M* (*SD*)** | **Range** | **ω** | |  | |  |
| **Heritage Cultural Identity Exploration** | **T1** | | 906 | 2.62 (0.58) | 1-4 | | .79 | | 490 | 2.35 (0.99) | 1-4 | .84 | | .34 [.22,0.46] | |  |
|  | **T2** | | 853 | 2.70 (0.55) | 1-4 | | .81 | | 465 | 2.44 (1.05) | 1-4 | .89 | | .32 [.19, .45] | |  |
|  | **T3** | | 844 | 2.63 (0.57) | 1-4 | | .83 | | 397 | 2.31 (1.02) | 1-4 | .88 | | .38 [.25, .52] | |  |
| **Heritage Cultural Identity Resolution** | **T1** | | 906 | 2.84 (0.67) | 1-4 | | .85 | | 491 | 3.16 (0.86) | 1-4 | .76 | | -.43 [-.55, -.31] | |  |
|  | **T2** | | 853 | 2.89 (0.64) | 1-4 | | .85 | | 466 | 3.23 (0.84) | 1-4 | .88 | | -.46 [-.60, -.34] | |  |
|  | **T3** | | 844 | 2.84 (0.65) | 1-4 | | .86 | | 397 | 3.17 (0.86) | 1-4 | .91 | | -.43 [-.56, -.30] | |  |
| **Heritage and Intercultural Learning** | **T1** | | 893 | 2.39 (0.71) | 1-5 | | .85 | | 497 | 2.92 (0.95) | 1-5 | .83 | | -.63 [-.75, -.52] | |  |
|  | **T2** | | 839 | 2.71 (0.61) | 1.14-5 | | .86 | | 459 | 3.04 (0.87) | 1-5 | .87 | | -.45 [-.57, -.32] | |  |
|  | **T3** | | 833 | 2.40 (0.68) | 1-5 | | .84 | | 400 | 2.95 (0.97) | 1-5 | .88 | | -.65 [-.80, -.53] | |  |
| **Equal Treatment** | **T1** | | 893 | 4.18 (0.68) | 1.60-5 | | .71 | | 496 | 4.08 (0.86) | 1-5 | .72 | | .14 [.03, .24] | |  |
|  | **T2** | | 839 | 4.04 (0.80) | 1-5 | | .85 | | 458 | 3.98 (0.93) | 1-5 | .79 | | .08 [-.05, .18] | |  |
|  | **T3** | | 833 | 3.92 (0.74) | 1-5 | | .79 | | 397 | 3.91 (0.83) | 1-5 | .71 | | .01 [-.11, .13] | |  |
| **Critical consciousness** | **T1** | | 891 | 2.41 (0.69) | 1-5 | | .72 | | 493 | 2.55 (1.01) | 1-5 | .76 | | -.16 [-.26, -.03] | |  |
|  | **T2** | | 839 | 2.44 (0.74) | 1-5 | | .78 | | 454 | 2.52 (0.97) | 1-5 | .77 | | -.09 [-.21, .04] | |  |
|  | **T3** | | 833 | 2.42 (0.74) | 1-5 | | .82 | | 397 | 2.63 (0.99) | 1-5 | .82 | | -.24 [-.37, -.11] | |  |

*Note.* Table describes size of sample, mean, standard deviation and range of mean for main study variables at T1, T2, and T3 for the Italian and German sample. Mean differences between the Italian and German sample are described with Cohen’s *d*, in which 0.2, 0.5, 0.8 denote small, medium and large effects, respectively (Cohen, 1988).

# Online Resource 4. *Differences of main study variables between time points in Italy.*

|  | **Time point** | ***N*** | ***M* (*SD*)** | **Time point** | ***N*** | ***M* (*SD*)** | **Cohen’s *d***  **[95% - CI** |
| --- | --- | --- | --- | --- | --- | --- | --- |
| **Heritage Cultural Identity Exploration** | T1 | 906 | 2.62 (0.58) | T2 | 853 | 2.70 (0.55) | -.14 [-.24, -.05] |
|  | T1 | 906 | 2.62 (0.58) | T3 | 844 | 2.63 (0.57) | -.01 [-.11, .08] |
|  | T2 | 853 | 2.70 (0.55) | T3 | 844 | 2.63 (0.57) | .13 [.04, .23] |
| **Heritage Cultural Identity Resolution** | T1 | 906 | 2.84 (0.67) | T2 | 853 | 2.89 (0.64) | -.08 [-.18, .01] |
|  | T1 | 906 | 2.84 (0.67) | T3 | 844 | 2.84 (0.65) | -.01 [-.11, .08] |
|  | T2 | 853 | 2.70 (0.55) | T3 | 844 | 2.84 (0.65) | .07 [-.02, .17] |
| **Heritage and Intercultural Learning** | T1 | 893 | 2.39 (0.71) | T2 | 839 | 2.71 (0.61) | -.48 [-.58, -.39] |
|  | T1 | 893 | 2.39 (0.71) | T3 | 833 | 2.40 (0.68) | -.02 [-.11, .08] |
|  | T2 | 839 | 2.71 (0.61) | T3 | 833 | 2.40 (0.68) | .48 [.38, .57] |
| **Equal Treatment** | T1 | 893 | 4.18 (0.68) | T2 | 839 | 4.04 (0.80) | .19 [.09, .28] |
|  | T1 | 893 | 4.18 (0.68) | T3 | 833 | 3.92 (0.74) | .37 [.27, .46] |
|  | T2 | 839 | 4.04 (0.80) | T3 | 833 | 3.92 (0.74) | .16 [.06, .25] |
| **Critical consciousness** | T1 | 891 | 2.41 (0.69) | T2 | 839 | 2.44 (0.74) | -.04 [-.14, .05] |
|  | T1 | 891 | 2.41 (0.69) | T3 | 833 | 2.42 (0.74) | -.01 [-.10, .09] |
|  | T2 | 839 | 2.44 (0.74) | T3 | 833 | 2.42 (0.74) | .03 [-.06, .13] |

*Note.* Table describes size of sample, mean, standard deviation and range of mean for main study variables at T1, T2, and T3 for the Italian sample. Mean differences between the time points are described with Cohen’s *d*, in which 0.2, 0.5, 0.8 denote small, medium and large effects, respectively (Cohen, 1988).

# Online Resource 5. *Differences of main study variables per time point Germany.*

|  | **Time point** | ***N*** | ***M* (*SD*)** | **Time point** | ***N*** | ***M* (*SD*)** | **Cohen’s *d***  **[95% - CI** |
| --- | --- | --- | --- | --- | --- | --- | --- |
| **Heritage Cultural Identity Exploration** | T1 | 490 | 2.35 (0.99) | T2 | 465 | 2.44 (1.05) | -.09 [-.21, .04] |
|  | T1 | 490 | 2.35 (0.99) | T3 | 397 | 2.31 (1.02) | .04 [-.10, .17] |
|  | T2 | 465 | 2.44 (1.05) | T3 | 397 | 2.31 (1.02) | .12 [-.01, .26] |
| **Heritage Cultural Identity Resolution** | T1 | 491 | 3.16 (0.86) | T2 | 466 | 3.23 (0.84) | -.08 [-.21, .05] |
|  | T1 | 491 | 3.16 (0.86) | T3 | 397 | 3.17 (0.86) | -.01 [-.14, .13] |
|  | T2 | 466 | 3.23 (0.84) | T3 | 397 | 3.17 (0.86) | .08 [-.06, .21] |
| **Heritage and Intercultural Learning** | T1 | 497 | 2.92 (0.95) | T2 | 459 | 3.04 (0.87) | -.14 [-.26, -.01] |
|  | T1 | 497 | 2.92 (0.95) | T3 | 400 | 2.95 (0.97) | -.03 [-.16, .11] |
|  | T2 | 459 | 3.04 (0.87) | T3 | 400 | 2.95 (0.97) | .11 [-.03, .24] |
| **Equal Treatment** | T1 | 496 | 4.08 (0.86) | T2 | 458 | 3.98 (0.93) | .11 [-.02, .24] |
|  | T1 | 496 | 4.08 (0.86) | T3 | 397 | 3.91 (0.83) | .19 [.06, .33] |
|  | T2 | 458 | 3.98 (0.93) | T3 | 397 | 3.91 (0.83) | .07 [-.06, .21] |
| **Critical consciousness** | T1 | 493 | 2.55 (1.01) | T2 | 454 | 2.52 (0.97) | .03 [-.10, .16] |
|  | T1 | 493 | 2.55 (1.01) | T3 | 397 | 2.63 (0.99) | -.08 [-.21, .05] |
|  | T2 | 454 | 2.52 (0.97) | T3 | 397 | 2.63 (0.99) | -.12 [-.25, .02] |

*Note.* Table describes size of sample, mean, standard deviation and range of mean for main study variables at T1, T2, and T3 for the German sample. Mean differences between the time points are described with Cohen’s *d*, in which 0.2, 0.5, 0.8 denote small, medium and large effects, respectively (Cohen, 1988).

# Online Resource 6. *Descriptives of main study variables for Italy, comparing students of immigrant descent and non-immigrant descent..*

|  |  | | **Immigrant Descent** | | |  | | | **Non-immigrant descent** | | | |  | | **Cohen’s *d*** | |
| --- | --- | --- | --- | --- | --- | --- | --- | --- | --- | --- | --- | --- | --- | --- | --- | --- |
|  |  | ***N*** | | ***M* (*SD*)** | **Range** | | **ω** | ***N*** | | ***M* (*SD*)** | **Range** | **ω** | | **[95% - CI]** | |  |
| **Heritage Cultural Identity Exploration** | **T1** | 291 | | 2.77 (0.63) | 1.14-4 | | .79 | 615 | | 2.55 (0.55) | 1-4 | .78 | | -.38 [-.52, -.24] | |  |
|  | **T2** | 268 | | 2.80 (0.61) | 1-4 | | .84 | 585 | | 2.66 (0.51) | 1-4 | .78 | | -.27 [-.41, -.12] | |  |
|  | **T3** | 261 | | 2.77 (0.65) | 1-4 | | .85 | 583 | | 2.57 (0.52) | 1-4 | .81 | | -.35 [-.50, -.21] | |  |
| **Heritage Cultural Identity Resolution** | **T1** | 291 | | 3.04 (0.69) | 1-4 | | .85 | 615 | | 2.74 (0.63) | 1-4 | .83 | | -.48 [-.61, -.33] | |  |
|  | **T2** | 268 | | 3.03 (0.67) | 1-4 | | .86 | 585 | | 2.82 (0.62) | 1-4 | .84 | | -.32 [-.47, -.18] | |  |
|  | **T3** | 261 | | 3.00 (0.68) | 1-4 | | .86 | 583 | | 2.77 (0.62) | 1-4 | .86 | | -.37 [-.52, -.22] | |  |
| **Heritage and Intercultural Learning** | **T1** | 286 | | 2.26 (0.78) | 1-5 | | .88 | 607 | | 2.45 (0.66) | 1-5 | .82 | | .26 [.12, .40] | |  |
|  | **T2** | 265 | | 2.59 (0.65) | 1.29-4.71 | | .88 | 574 | | 2.76 (0.58) | 1.14-5 | .84 | | .28 [.13, .42] | |  |
|  | **T3** | 257 | | 2.28 (0.72) | 1-4.29 | | .85 | 576 | | 2.45 (0.65) | 1-5 | .83 | | .26 [.11, .41] | |  |
| **Equal Treatment** | **T1** | 286 | | 4.13 (0.72) | 1.60-5 | | .74 | 607 | | 4.21 (0.66) | 1.6-5 | .70 | | .12 [-.02, .26] | |  |
|  | **T2** | 265 | | 4.01 (0.82) | 1.40-5 | | .84 | 574 | | 4.06 (0.79) | 1-5 | .85 | | .06 [-.09, .20] | |  |
|  | **T3** | 257 | | 3.95 (0.77) | 1.60-5 | | .80 | 576 | | 3.91 (0.72) | 1-5 | .78 | | -.06 [-.21, .09] | |  |
| **Critical consciousness** | **T1** | 286 | | 2.31 (0.73) | 1-5 | | .75 | 605 | | 2.46 (0.67) | 1-5 | .70 | | .22 [.08, .36] | |  |
|  | **T2** | 265 | | 2.33 (0.77) | 1-5 | | .79 | 574 | | 2.50 (0.72) | 1-5 | .78 | | .23 [.08, .37] | |  |
|  | **T3** | 257 | | 2.34 (0.74) | 1-5 | | .82 | 576 | | 2.45 (0.74) | 1-5 | .82 | | .16 [.01, .31] | |  |

*Note.* Table describes size of sample, mean, standard deviation and range of mean for main study variables at T1, T2, and T3 for the Italian sample, comparing students of immigrant and non-immigrant descent. Mean differences between the subsamples are described with Cohen’s *d*, in which 0.2, 0.5, 0.8 denote small, medium and large effects, respectively (Cohen, 1988).

# Online Resource 7. *Descriptives of main study variables for Germany, comparing students of immigrant descent and non-immigrant descent..*

|  |  | | **Immigrant Descent** | | |  | | | **Non-immigrant descent** | | | |  | | **Cohen’s *d*** | |
| --- | --- | --- | --- | --- | --- | --- | --- | --- | --- | --- | --- | --- | --- | --- | --- | --- |
|  |  | ***N*** | | ***M* (*SD*)** | **Range** | | **ω** | ***N*** | | ***M* (*SD*)** | **Range** | **ω** | | **[95% - CI]** | |  |
| **Heritage Cultural Identity Exploration** | **T1** | 223 | | 2.06 (0.86) | 1-4 | | .84 | 264 | | 2.59 (1.04) | 1-4 | .81 | | -.55 [-.74, -.37] | |  |
|  | **T2** | 188 | | 2.16 (0.99) | 1-4 | | .87 | 254 | | 2.69 (1.02) | 1-4 | .91 | | -.53 [-.72, -.34] | |  |
|  | **T3** | 178 | | 2.01 (0.87) | 1-4 | | .87 | 201 | | 2.62 (1.05) | 1-4 | .86 | | -.63 [-.83, -.42] | |  |
| **Heritage Cultural Identity Resolution** | **T1** | 223 | | 2.81 (0.88) | 1-4 | | .68 | 265 | | 3.45 (0.73) | 1-4 | .87 | | -.81 [-.99, -.62] | |  |
|  | **T2** | 188 | | 2.88 (0.86) | 1-4 | | .84 | 255 | | 3.50 (0.69) | 1-4 | .88 | | -.81 [-1.00, -.61] | |  |
|  | **T3** | 178 | | 2.85 (0.88) | 1-4 | | .86 | 201 | | 3.48 (0.68) | 1-4 | .92 | | -.81 [-1.02, -.60] | |  |
| **Heritage and Intercultural Learning** | **T1** | 226 | | 3.01 (0.93) | 1-5 | | .82 | 268 | | 2.84 (0.97) | 1-5 | .85 | | .18 [.01, .36] | |  |
|  | **T2** | 186 | | 3.15 (0.83) | 1-5 | | .86 | 252 | | 2.98 (0.89) | 1-5 | .88 | | .19 [-.00, .38] | |  |
|  | **T3** | 180 | | 3.11 (0.85) | 1-5 | | .87 | 203 | | 2.86 (1.02) | 1-5 | .88 | | .26 [.06, .46] | |  |
| **Equal Treatment** | **T1** | 226 | | 4.20 (0.73) | 1-5 | | .73 | 267 | | 3.98 (0.94) | 1-5 | .66 | | .27 [.09, .45] | |  |
|  | **T2** | 186 | | 4.17 (0.80) | 1-5 | | .77 | 251 | | 3.84 (0.98) | 1-5 | .79 | | .36 [.17, .55] | |  |
|  | **T3** | 180 | | 3.99 (0.76) | 1-5 | | .67 | 200 | | 3.83 (0.85) | 1-5 | .74 | | .20 [.00, .41] | |  |
| **Critical consciousness** | **T1** | 224 | | 2.72 (0.98) | 1-5 | | .73 | 266 | | 2.40 (1.00) | 1-5 | .78 | | .33 [.15, .51] | |  |
|  | **T2** | 185 | | 2.72 (0.96) | 1-5 | | .70 | 250 | | 2.41 (0.95) | 1-5 | .82 | | .32 [.13, .52] | |  |
|  | **T3** | 180 | | 2.92 (0.89) | 1-5 | | .80 | 200 | | 2.45 (1.00) | 1-5 | .80 | | .49 [.29, .70] | |  |

*Note.* Table describes size of sample, mean, standard deviation and range of mean for main study variables at T1, T2, and T3 for the German sample, comparing students of immigrant and non-immigrant descent. Mean differences between the subsamples are described with Cohen’s *d*, in which 0.2, 0.5, 0.8 denote small, medium and large effects, respectively (Cohen, 1988).

# Online Resource 8. *Measurement invariance Italian sample, comparing students of immigrant descent and non-immigrant descent.*

|  | **n. par.** | **χ²** | **df** | **Δχ²** | **Δdf** | **p-value** | **CFI** | **ΔCFI** | **RMSEA** |
| --- | --- | --- | --- | --- | --- | --- | --- | --- | --- |
| **Heritage Cultural Identity Exploration** |  |  |  |  |  |  |  |  |  |
| **T1** |  |  |  |  |  |  |  |  |  |
| Configural invariance model | 42 | 301.630 | 28 |  |  |  | 0.842 |  | 0.147 |
| Metric invariance model | 42 | 312.990 | 34 | 11.361 | 6 | 0.078 | 0.839 | -0.003 | 0.135 |
| Scalar invariance model | 43 | 339.440 | 40 | 37.814 | 12 | 0.000 | 0.827 | -0.015 | 0.129 |
| Strict invariance model | 43 | 378.400 | 47 | 76.768 | 19 | 0.000 | 0.809 | -0.033 | 0.125 |
| **T2** |  |  |  |  |  |  |  |  |  |
| Configural invariance model | 42 | 301.210 | 28 |  |  |  | 0.854 |  | 0.152 |
| Metric invariance model | 42 | 303.840 | 34 | 2.626 | 6 | 0.854 | 0.856 | 0.002 | 0.137 |
| Scalar invariance model | 43 | 313.880 | 40 | 12.669 | 12 | 0.394 | 0.854 | 0.000 | 0.127 |
| Strict invariance model | 43 | 325.510 | 47 | 24.296 | 19 | 0.185 | 0.851 | -0.003 | 0.118 |
| **T3** |  |  |  |  |  |  |  |  |  |
| Configural invariance model | 42 | 209.760 | 28 |  |  |  | 0.913 |  | 0.124 |
| Metric invariance model | 42 | 212.350 | 34 | 2.586 | 6 | 0.859 | 0.914 | 0.002 | 0.112 |
| Scalar invariance model | 43 | 218.830 | 40 | 9.07 | 12 | 0.697 | 0.914 | 0.001 | 0.103 |
| Strict invariance model | 43 | 257.710 | 47 | 47.944 | 19 | 0.000 | 0.899 | -0.014 | 0.103 |
| **Heritage Cultural Identity Resolution** |  |  |  |  |  |  |  |  |  |
| **T1** |  |  |  |  |  |  |  |  |  |
| Configural invariance model | 24 | 9.485 | 4 |  |  |  | 0.996 |  | 0.055 |
| Metric invariance model | 24 | 9.663 | 7 | 0.178 | 3 | 0.981 | 0.998 | 0.002 | 0.029 |
| Scalar invariance model | 25 | 10.466 | 10 | 0.981 | 6 | 0.986 | 1.000 | 0.003 | 0.010 |
| Strict invariance model | 25 | 17.165 | 14 | 7.680 | 10 | 0.660 | 0.998 | 0.002 | 0.022 |
| **T2** |  |  |  |  |  |  |  |  |  |
| Configural invariance model | 24 | 5.862 | 4 |  |  |  | 0.999 |  | 0.033 |
| Metric invariance model | 24 | 8.035 | 7 | 2.173 | 3 | 0.537 | 0.999 | 0.001 | 0.019 |
| Scalar invariance model | 25 | 8.142 | 10 | 2.279 | 6 | 0.892 | 1.000 | 0.001 | 0.000 |
| Strict invariance model | 25 | 14.693 | 14 | 8.830 | 10 | 0.548 | 1.000 | 0.001 | 0.011 |
| **T3** |  |  |  |  |  |  |  |  |  |
| Configural invariance model | 24 | 7.997 | 4 |  |  |  | 0.997 |  | 0.049 |
| Metric invariance model | 24 | 9.737 | 7 | 1.741 | 3 | 0.628 | 0.998 | 0.001 | 0.030 |
| Scalar invariance model | 25 | 11.709 | 10 | 3.772 | 6 | 0.716 | 0.999 | 0.002 | 0.020 |
| Strict invariance model | 25 | 28.187 | 14 | 20.190* | 10 | 0.028 | 0.991 | -0.007 | 0.049 |
| **Heritage and Intercultural Learning** |  |  |  |  |  |  |  |  |  |
| **T1** |  |  |  |  |  |  |  |  |  |
| Configural invariance model | 42 | 115.100 | 28 |  |  |  | 0.957 |  | 0.084 |
| Metric invariance model | 42 | 125.340 | 34 | 10.244 | 6 | 0.115 | 0.955 | -.002 | 0.078 |
| Scalar invariance model | 43 | 139.780 | 40 | 24.685 | 12 | 0.016 | 0.951 | -.006 | 0.075 |
| Strict invariance model | 43 | 147.420 | 47 | 32.325 | 19 | 0.029 | 0.951 | -.007 | 0.070 |
| **T2** |  |  |  |  |  |  |  |  |  |
| Configural invariance model | 42 | 59.767 | 28 |  |  |  | 0.985 |  | 0.052 |
| Metric invariance model | 42 | 64.871 | 34 | 5.104 | 6 | 0.531 | 0.985 | 0.000 | 0.047 |
| Scalar invariance model | 43 | 68.342 | 40 | 8.575 | 12 | 0.739 | 0.986 | 0.002 | 0.041 |
| Strict invariance model | 43 | 77.017 | 47 | 17.250 | 19 | 0.573 | 0.985 | 0.001 | 0.039 |
| **T3** |  |  |  |  |  |  |  |  |  |
| Configural invariance model | 42 | 58.741 | 28 |  |  |  | 0.984 |  | 0.051 |
| Metric invariance model | 42 | 60.565 | 34 | 1.824 | 6 | 0.935 | 0.986 | 0.002 | 0.043 |
| Scalar invariance model | 43 | 76.987 | 40 | 18.246 | 12 | 0.108 | 0.981 | -0.003 | 0.047 |
| Strict invariance model | 43 | 87.338 | 47 | 28.598 | 19 | 0.073 | 0.979 | -0.005 | 0.046 |
| **Equal Treatment** |  |  |  |  |  |  |  |  |  |
| **T1** |  |  |  |  |  |  |  |  |  |
| Configural invariance model | 30 | 71.905 | 10 |  |  |  | 0.917 |  | 0.118 |
| Metric invariance model | 30 | 81.483 | 14 | 9.577 | 4 | 0.048 | 0.909 | -0.008 | 0.104 |
| Scalar invariance model | 31 | 96.554 | 18 | 24.649 | 8 | 0.002 | 0.894 | -0.022 | 0.099 |
| Strict invariance model | 31 | 106.353 | 23 | 34.448 | 13 | 0.001 | 0.888 | -0.029 | 0.090 |
| **T2^a^** |  |  |  |  |  |  |  |  |  |
| Configural invariance model | 24 | 15.550 | 4 |  |  |  | 0.985 |  | 0.083 |
| Metric invariance model | 24 | 22.435 | 7 | 6.885 | 3 | 0.076 | 0.980 | -0.005 | 0.073 |
| Scalar invariance model | 25 | 26.460 | 10 | 10.911 | 6 | 0.091 | 0.978 | -0.006 | 0.063 |
| Strict invariance model | 25 | 32.888 | 14 | 17.338 | 10 | 0.067 | 0.975 | -0.010 | 0.057 |
| **T3** |  |  |  |  |  |  |  |  |  |
| Configural invariance model | 30 | 6.785 | 10 |  |  |  | 1.000 |  | 0.000 |
| Metric invariance model | 30 | 11.034 | 14 | 4.249 | 4 | 0.373 | 1.000 | 0.000 | 0.000 |
| Scalar invariance model | 31 | 30.997 | 18 | 24.212** | 8 | 0.002 | 0.989 | -0.011 | 0.042 |
| Strict invariance model | 31 | 40.193 | 23 | 33.408** | 13 | 0.001 | 0.985 | -0.015 | 0.042 |
| **Critical consciousness** |  |  |  |  |  |  |  |  |  |
| **T1** |  |  |  |  |  |  |  |  |  |
| Configural invariance model | 30 | 56.398 | 10 |  |  |  | 0.941 |  | 0.102 |
| Metric invariance model | 30 | 58.159 | 14 | 1.760 | 4 | 0.780 | 0.944 | 0.003 | 0.084 |
| Scalar invariance model | 31 | 65.135 | 18 | 8.737 | 8 | 0.365 | 0.940 | -0.001 | 0.077 |
| Strict invariance model | 31 | 74.207 | 23 | 17.808 | 13 | 0.165 | 0.935 | -0.006 | 0.071 |
| **T2** |  |  |  |  |  |  |  |  |  |
| Configural invariance model | 30 | 26.482 | 10 |  |  |  | 0.984 |  | 0.063 |
| Metric invariance model | 30 | 31.873 | 14 | 5.391 | 4 | 0.250 | 0.982 | -0.001 | 0.055 |
| Scalar invariance model | 31 | 37.008 | 18 | 10.526 | 8 | 0.230 | 0.981 | -0.002 | 0.050 |
| Strict invariance model | 31 | 44.238 | 23 | 17.756 | 13 | 0.167 | 0.979 | -0.005 | 0.047 |
| **T3** |  |  |  |  |  |  |  |  |  |
| Configural invariance model | 30 | 47.334 | 10 |  |  |  | 0.972 |  | 0.095 |
| Metric invariance model | 30 | 51.207 | 14 | 3.873 | 4 | 0.423 | 0.972 | 0.000 | 0.080 |
| Scalar invariance model | 31 | 53.595 | 18 | 6.261 | 8 | 0.618 | 0.973 | 0.001 | 0.069 |
| Strict invariance model | 31 | 56.969 | 23 | 9.636 | 13 | 0.723 | 0.975 | 0.003 | 0.060 |

*Note.* All models are compared to the configural model as the baseline for assessing model fit. ^a^The analysis encountered difficulties due to non-positive-definite sample covariance matrices, which prevented the estimation of invariances. As a result, one item was removed from the dataset to address this issue*.* n. par. = number of parameters, χ2 = Model chi-square, df = Degrees of freedom, ∆χ2 = Chi-squared difference test, CFI = Comparative Fit Index, ∆CFI = CFI differences, RMSEA = Root Mean Square Error of Approximation.

# Online Resource 9. *Measurement invariance German sample, comparing students of immigrant descent and non-immigrant descent.*

|  | **n. par.** | **χ²** | **df** | **Δχ²** | **Δdf** | **p-value** | **CFI** | **ΔCFI** | **RMSEA** |
| --- | --- | --- | --- | --- | --- | --- | --- | --- | --- |
| **Heritage Cultural Identity Exploration** |  |  |  |  |  |  |  |  |  |
| **T1** |  |  |  |  |  |  |  |  |  |
| Configural invariance model | 18 | 0.000 | 0 |  |  |  | 1.000 |  | 0.000 |
| Metric invariance model | 18 | 4.412 | 2 | 4.412 | 2 | 0.110 | 0.995 | -0.005 | 0.071 |
| Scalar invariance model | 19 | 10.165 | 4 | 10.165 | 4 | 0.038 | 0.988 | -0.012 | 0.080 |
| Strict invariance model | 19 | 18.232 | 7 | 18.232 | 7 | 0.011 | 0.979 | -0.021 | 0.082 |
| **T2** |  |  |  |  |  |  |  |  |  |
| Configural invariance model | 18 | 0.000 | 0 |  |  |  | 1.000 |  | 0.000 |
| Metric invariance model | 18 | 0.461 | 2 | 0.461 | 2 | 0.794 | 1.000 | 0.000 | 0.000 |
| Scalar invariance model | 19 | 5.969 | 4 | 5.969 | 4 | 0.202 | 0.997 | -0.003 | 0.048 |
| Strict invariance model | 19 | 21.893 | 7 | 21.893 | 7 | 0.002 | 0.979 | -0.021 | 0.099 |
| **T3** |  |  |  |  |  |  |  |  |  |
| Configural invariance model | 18 | 0.000 | 0 |  |  |  | 1.000 |  | 0.000 |
| Metric invariance model | 18 | 3.664 | 2 | 2.664 | 2 | 0.160 | 0.997 | -0.003 | 0.067 |
| Scalar invariance model | 19 | 3.984 | 4 | 3.984 | 4 | 0.408 | 1.000 | 0.000 | 0.000 |
| Strict invariance model | 19 | 14.821 | 7 | 14.821 | 7 | 0.038 | 0.986 | -0.014 | 0.077 |
| **Heritage Cultural Identity Resolution** |  |  |  |  |  |  |  |  |  |
| **T1** |  |  |  |  |  |  |  |  |  |
| Configural invariance model | 18 | 0.000 | 0 |  |  |  | 1.000 |  | 0.000 |
| Metric invariance model | 18 | 1.747 | 2 | 1.747 | 2 | 0.418 | 1.000 | 0.000 | 0.000 |
| Scalar invariance model | 19 | 5.864 | 4 | 5.864 | 4 | 0.210 | 0.996 | -0.004 | 0.044 |
| Strict invariance model | 19 | 350.500 | 7 | 350.000 | 7 | 0.000 | 0.279 | -0.721 | 0.453 |
| **T2** |  |  |  |  |  |  |  |  |  |
| Configural invariance model | 18 | 0.000 | 0 |  |  |  | 1.000 |  | 0.000 |
| Metric invariance model | 18 | 4.102 | 2 | 4.102 | 2 | 0.129 | 0.996 | -0.004 | 0.070 |
| Scalar invariance model | 19 | 4.368 | 4 | 4.368 | 4 | 0.359 | 0.999 | -0.001 | 0.021 |
| Strict invariance model | 19 | 7.782 | 7 | 7.782 | 7 | 0.352 | 0.999 | -0.001 | 0.023 |
| **T3** |  |  |  |  |  |  |  |  |  |
| Configural invariance model | 18 | 0.000 | 0 |  |  |  | 1.000 |  | 0.000 |
| Metric invariance model | 18 | 4.517 | 2 | 4.517 | 2 | 0.105 | 0.996 | -0.004 | 0.082 |
| Scalar invariance model | 19 | 7.540 | 4 | 7.540 | 4 | 0.110 | 0.995 | -0.005 | 0.069 |
| Strict invariance model | 19 | 17.258 | 7 | 17.258 | 7 | 0.016 | 0.984 | -0.016 | 0.088 |
| **Heritage and Intercultural Learning** |  |  |  |  |  |  |  |  |  |
| **T1** |  |  |  |  |  |  |  |  |  |
| Configural invariance model | 42 | 57.330 | 28 |  |  |  | 0.968 |  | 0.070 |
| Metric invariance model | 42 | 68.917 | 34 | 11.587 | 6 | 0.072 | 0.962 | -0.006 | 0.069 |
| Scalar invariance model | 43 | 77.681 | 40 | 20.351 | 12 | 0.061 | 0.960 | -0.009 | 0.066 |
| Strict invariance model | 43 | 102.730 | 47 | 45.396 | 19 | 0.001 | 0.940 | -0.028 | 0.074 |
| **T2** |  |  |  |  |  |  |  |  |  |
| Configural invariance model | 42 | 141.870 | 28 |  |  |  | 0.897 |  | 0.145 |
| Metric invariance model | 42 | 145.190 | 34 | 3.320 | 6 | 0.768 | 0.900 | 0.002 | 0.130 |
| Scalar invariance model | 43 | 146.710 | 40 | 4.841 | 12 | 0.963 | 0.904 | 0.006 | 0.117 |
| Strict invariance model | 43 | 185.540 | 47 | 43.669 | 19 | 0.001 | 0.875 | -0.022 | 0.123 |
| **T3** |  |  |  |  |  |  |  |  |  |
| Configural invariance model | 42 | 82.104 | 28 |  |  |  | 0.948 |  | 0.105 |
| Metric invariance model | 42 | 89.613 | 34 | 7.509 | 6 | 0.276 | 0.947 | -0.001 | 0.097 |
| Scalar invariance model | 43 | 104.009 | 40 | 21.906 | 12 | 0.039 | 0.939 | -0.009 | 0.095 |
| Strict invariance model | 43 | 172.425 | 47 | 90.321 | 19 | 0.000 | 0.880 | -0.068 | 0.123 |
| **Equal Treatment** |  |  |  |  |  |  |  |  |  |
| **T1** |  |  |  |  |  |  |  |  |  |
| Configural invariance model | 30 | 35.423 | 10 |  |  |  | 0.928 |  | 0.107 |
| Metric invariance model | 30 | 44.499 | 14 | 9.076 | 4 | 0.059 | 0.913 | -0.014 | 0.099 |
| Scalar invariance model | 31 | 56.980 | 18 | 21.557 | 8 | 0.006 | 0.889 | -0.039 | 0.098 |
| Strict invariance model | 31 | 94.459 | 23 | 59.037 | 13 | 0.000 | 0.797 | -0.131 | 0.118 |
| **T2^a^** |  |  |  |  |  |  |  |  |  |
| Configural invariance model | 24 | 8.975 | 4 |  |  |  | 0.970 |  | 0.079 |
| Metric invariance model | 24 | 12.484 | 7 | 3.509 | 3 | 0.320 | 0.967 | -0.003 | 0.062 |
| Scalar invariance model | 25 | 15.723 | 10 | 5.749 | 6 | 0.452 | 0.972 | 0.002 | 0.048 |
| Strict invariance model | 25 | 81.276 | 14 | 72.301 | 10 | 0.000 | 0.597 | -0.373 | 0.155 |
| **T3** | 30 | 25.290 | 10 |  |  |  | 0.949 |  | 0.093 |
| Configural invariance model | 30 | 29.229 | 14 | 3.939 | 4 | 0.414 | 0.949 | -0.015 | 0.079 |
| Metric invariance model | 31 | 46.584 | 18 | 21.304 | 8 | 0.006 | 0.904 | -0.044 | 0.095 |
| Scalar invariance model | 31 | 84.127 | 23 | 58.837 | 13 | 0.000 | 0.796 | -0.153 | 0.123 |
| Strict invariance model |  |  |  |  |  |  |  |  |  |
| **T1** |  |  |  |  |  |  |  |  |  |
| Configural invariance model | 30 | 6.470 | 10 |  |  |  | 1.000 |  | 0.000 |
| Metric invariance model | 30 | 12.47 | 14 | 6.000 | 4 | 0.1991 | 1.000 | 0.000 | 0.000 |
| Scalar invariance model | 31 | 22.291 | 18 | 15.821 | 8 | 0.045 | 0.990 | -0.010 | 0.033 |
| Strict invariance model | 31 | 46.999 | 23 | 40.530 | 13 | 0.000 | 0.944 | -0.056 | 0.070 |
| **T2** |  |  |  |  |  |  |  |  |  |
| Configural invariance model | 30 | 24.097 | 10 |  |  |  | 0.969 |  | 0.085 |
| Metric invariance model | 30 | 30.417 | 14 | 6.321 | 4 | 0.176 | 0.964 | -0.005 | 0.077 |
| Scalar invariance model | 31 | 37.375 | 18 | 13.2778 | 8 | 0.103 | 0.958 | -0.011 | 0.074 |
| Strict invariance model | 31 | 96.761 | 23 | 72.664 | 13 | 0.000 | 0.839 | -0.130 | 0.128 |
| **T3** |  |  |  |  |  |  |  |  |  |
| Configural invariance model | 30 | 26.199 | 10 |  |  |  | 0.967 |  | 0.096 |
| Metric invariance model | 30 | 31.855 | 14 | 5.656 | 4 | 0.226 | 0.963 | -0.003 | 0.085 |
| Scalar invariance model | 31 | 50.553 | 18 | 24.353** | 8 | 0.002 | 0.933 | -0.034 | 0.101 |
| Strict invariance model | 31 | 69.094 | 23 | 42.895*** | 13 | 0.000 | 0.905 | -0.062 | 0.106 |

*Note.* All models are compared to the configural model as the baseline for assessing model fit.. ^a^The analysis encountered difficulties due to non-positive-definite sample covariance matrices, which prevented the estimation of invariances. As a result, one item was removed from the dataset to address this issue. n. par. = number of parameters, χ2 = Model chi-square, df = Degrees of freedom, ∆χ2 = Chi-squared difference test, CFI = Comparative Fit Index, ∆CFI = CFI differences, RMSEA = Root Mean Square Error of Approximation*.*

# Online Resource 10. *Longitudinal measurement invariance Italian sample.*

|  | **n. par.** | **χ²** | **df** | **Δχ²** | **Δdf** | **p-value** | **CFI** | **ΔCFI** | **RMSEA** |
| --- | --- | --- | --- | --- | --- | --- | --- | --- | --- |
| **Heritage Cultural Identity Exploration** |  |  |  |  |  |  |  |  |  |
| Configural invariance model | 87 | 868.970 | 165 |  |  |  | 0.892 |  | 0.076 |
| Metric invariance model | 89 | 917.280 | 177 | 48.316 | 12 | 0.000 | 0.886 | -0.006 | 0.075 |
| Scalar invariance model | 91 | 1246.380 | 189 | 377.410 | 24 | 0.000 | 0.837 | -0.054 | 0.087 |
| Strict invariance model | 91 | 1465.840 | 203 | 596.870 | 38 | 0.000 | 0.805 | -0.086 | 0.092 |
| **Heritage Cultural Identity Resolution** |  |  |  |  |  |  |  |  |  |
| Configural invariance model | 51 | 69.366 | 39 |  |  |  | 0.994 |  | 0.032 |
| Metric invariance model | 53 | 75.511 | 45 | 6.145 | 6 | 0.407 | 0.994 | 0.000 | 0.030 |
| Scalar invariance model | 55 | 88.895 | 51 | 19.529 | 12 | 0.077 | 0.992 | -0.002 | 0.032 |
| Strict invariance model | 55 | 122.541 | 59 | 122.541 | 20 | 0.000 | 0.987 | -0.007 | 0.038 |
| **Heritage and Intercultural Learning** |  |  |  |  |  |  |  |  |  |
| Configural invariance model | 87 | 384.480 | 165 |  |  |  | 0.965 |  | 0.044 |
| Metric invariance model | 89 | 491.00 | 177 | 106.520 | 12 | 0.000 | 0.950 | -0.015 | 0.050 |
| Scalar invariance model | 91 | 624.370 | 189 | 239.890 | 24 | 0.000 | 0.931 | -0.034 | 0.057 |
| Strict invariance model | 91 | 767.520 | 203 | 383.030 | 38 | 0.000 | 0.911 | -0.054 | 0.063 |
| **Equal Treatment^a^** |  |  |  |  |  |  |  |  |  |
| Configural invariance model | 51 | 94.054 | 39 |  |  |  | 0.978 |  | 0.044 |
| Metric invariance model | 53 | 190.248 | 45 | 96.194 | 6 | 0.000 | 0.942 | -0.036 | 0.067 |
| Scalar invariance model | 55 | 372.392 | 51 | 278.340 | 12 | 0.000 | 0.871 | -0.107 | 0.094 |
| Strict invariance model | 55 | 429.635 | 59 | 335.580 | 20 | 0.000 | 0.851 | -0.127 | 0.094 |
| **Critical consciousness** |  |  |  |  |  |  |  |  |  |
| Configural invariance model | 63 | 178.560 | 72 |  |  |  | 0.970 |  | 0.046 |
| Metric invariance model | 65 | 198.750 | 80 | 20.191 | 8 | 0.010 | 0.967 | -0.003 | 0.046 |
| Scalar invariance model | 67 | 266.670 | 88 | 88.113 | 16 | 0.000 | 0.950 | -0.020 | 0.053 |
| Strict invariance model | 67 | 371.510 | 98 | 192.960 | 26 | 0.000 | 0.923 | -0.047 | 0.063 |

*Note.* All models are compared to the configural model as the baseline for assessing model fit. n. par. = number of parameters, χ2 = Model chi-square, df = Degrees of freedom, ∆χ2 = Chi-squared difference test, CFI = Comparative Fit Index, ∆CFI = CFI differences, RMSEA = Root Mean Square Error of Approximation*.*

# Online Resource 11. *Longitudinal measurement invariance German sample.*

|  | **n. par.** | **χ²** | **df** | **Δχ²** | **Δdf** | **p-value** | **CFI** | **ΔCFI** | **RMSEA** |
| --- | --- | --- | --- | --- | --- | --- | --- | --- | --- |
| **Heritage Cultural Identity Exploration** |  |  |  |  |  |  |  |  |  |
| Configural invariance model | 39 | 46.510 | 15 |  |  |  | 0.982 |  | 0.090 |
| Metric invariance model | 41 | 49.425 | 19 | 2.915 | 4 | 0.572 | 0.982 | 0.001 | 0.078 |
| Scalar invariance model | 43 | 50.744 | 23 | 4.234 | 8 | 0.836 | 0.984 | 0.002 | 0.068 |
| Strict invariance model | 43 | 74.260 | 29 | 27.750 | 14 | 0.015 | 0.974 | -0.008 | 0.077 |
| **Heritage Cultural Identity Resolution** |  |  |  |  |  |  |  |  |  |
| Configural invariance model | 39 | 28.130 | 15 |  |  |  | 0.991 |  | 0.057 |
| Metric invariance model | 41 | 30.732 | 19 | 2.602 | 4 | 0.626 | 0.992 | 0.001 | 0.048 |
| Scalar invariance model | 43 | 31.473 | 23 | 3.343 | 8 | 0.911 | 0.994 | 0.003 | 0.037 |
| Strict invariance model | 43 | 677.550 | 29 | 649.42 | 14 | 0.000 | 0.557 | -0.434 | 0.289 |
| **Heritage and Intercultural Learning** |  |  |  |  |  |  |  |  |  |
| Configural invariance model | 87 | 324.650 | 165 |  |  |  | 0.929 |  | 0.067 |
| Metric invariance model | 89 | 348.660 | 177 | 24.016 | 12 | 0.020 | 0.924 | -0.005 | 0.067 |
| Scalar invariance model | 91 | 404.820 | 189 | 80.176 | 24 | 0.000 | 0.904 | -0.025 | 0.072 |
| Strict invariance model | 91 | 442.100 | 203 | 117.460 | 38 | 0.000 | 0.894 | -0.035 | 0.074 |
| **Equal Treatment^a^** |  |  |  |  |  |  |  |  |  |
| Configural invariance model | 51 | 58.185 | 39 |  |  |  | 0.963 |  | 0.045 |
| Metric invariance model | 53 | 66.405 | 45 | 8.219 | 6 | 0.223 | 0.959 | -0.004 | 0.045 |
| Scalar invariance model | 55 | 69.129 | 51 | 10.943 | 12 | 0.534 | 0.965 | 0.002 | 0.039 |
| Strict invariance model | 55 | 84.194 | 59 | 26.009 | 20 | 0.166 | 0.952 | -0.012 | 0.042 |
| **Critical consciousness** |  |  |  |  |  |  |  |  |  |
| Configural invariance model | 63 | 91.014 | 72 |  |  |  | 0.982 |  | 0.034 |
| Metric invariance model | 65 | 96.310 | 80 | 5.296 | 8 | 0.736 | 0.985 | 0.003 | 0.030 |
| Scalar invariance model | 67 | 123.361 | 88 | 32.348 | 16 | 0.009 | 0.967 | -0.015 | 0.042 |
| Strict invariance model | 67 | 157.918 | 98 | 66.904 | 26 | 0.000 | 0.944 | -0.038 | 0.052 |

*Note.* All models are compared to the configural model as the baseline for assessing model fit. ^a^The analysis encountered difficulties due to non-positive-definite sample covariance matrices, which prevented the estimation of invariances. As a result, one item was removed from the dataset to address this issue. n. par. = number of parameters, χ2 = Model chi-square, df = Degrees of freedom, ∆χ2 = Chi-squared difference test, CFI = Comparative Fit Index, ∆CFI = CFI differences, RMSEA = Root Mean Square Error of Approximation*.*

# Online Resource 12. *Correlations main study variables and controls – Italy.*

|  | **2.** | | **3.** | | **4.** | | **5.** | | **6.** | | **7.** | | **8.** | | **9.** | | **10.** | | **11.** | | **12.** | | **13.** | | **14.** | | **15.** | | **16.** | | **17.** | | **18.** | | **19.** | |
| --- | --- | --- | --- | --- | --- | --- | --- | --- | --- | --- | --- | --- | --- | --- | --- | --- | --- | --- | --- | --- | --- | --- | --- | --- | --- | --- | --- | --- | --- | --- | --- | --- | --- | --- | --- | --- |
| 1. **Intervention** | .01 | | -.07 | | -.04 | | -.03 | | -.07 | | .01 | | -.05 | | .08 | | .03 | | -.03 | | .03 | | .00 | | .02 | | -.07 | | -.08 | | -.03 | | .05 | | .02 | |
| 1. **Age** |  | | -.12 | | .20 | | .04 | | .02 | | .01 | | .08 | | .07 | | .04 | | -.02 | | -.02 | | -.01 | | -.05 | | -.02 | | -.06 | | -.07 | | -.07 | | -.03 | |
| 1. **Gender** |  | |  | | -.01 | | .06 | | .06 | | .05 | | -.06 | | -.06 | | -.03 | | .02 | | .02 | | -.12 | | .06 | | .20 | | .18 | | .08 | | -.03 | | -.04 | |
| 1. **Immigrant descent** |  | |  | |  | | .18 | | .12 | | .16 | | .22 | | .15 | | .17 | | -.12 | | -.13 | | -.12 | | -.06 | | -.03 | | .03 | | -.10 | | -.10 | | -.07 | |
| 1. **Exploration T1** |  | |  | |  | |  | | .60 | | .58 | | .50 | | .39 | | .41 | | .16 | | .16 | | .12 | | -.02 | | -.07 | | -.06 | | .06 | | .06 | | .09 | |
| 1. **Exploration T2** |  | |  | |  | |  | |  | | .70 | | .40 | | .61 | | .51 | | .14 | | .20 | | .10 | | -.04 | | -.04 | | -.03 | | .08 | | .11 | | .10 | |
| 1. **Exploration T3** |  | |  | |  | |  | |  | |  | | .36 | | .42 | | .58 | | .16 | | .20 | | .18 | | -.11 | | -.07 | | -.08 | | .08 | | .10 | | .14 | |
| 1. **Resolution T1** |  | |  | |  | |  | |  | |  | |  | | .56 | | .54 | | .19 | | .08 | | .14 | | -.12 | | -.08 | | -.14 | | .11 | | .02 | | .12 | |
| 1. **Resolution T2** |  | |  | |  | |  | |  | |  | |  | |  | | .68 | | .17 | | .18 | | .13 | | -.03 | | -.04 | | -.04 | | .11 | | .11 | | .11 | |
| 1. **Resolution T3** |  | |  | |  | |  | |  | |  | |  | |  | |  | | .14 | | .14 | | .13 | | -.02 | | .01 | | -.02 | | .05 | | .06 | | .11 | |
| 1. **Heritage Learning T1** |  | |  | |  | |  | |  | |  | |  | |  | |  | |  | | .58 | | .57 | | -.23 | | -.12 | | -.26 | | .62 | | .45 | | .45 | |
| 1. **Heritage Learning T2** |  | |  | |  | |  | |  | |  | |  | |  | |  | |  | |  | | .64 | | -.04 | | -.13 | | -.21 | | .42 | | .65 | | .51 | |
| 1. **Heritage Learning T3** |  | |  | |  | |  | |  | |  | |  | |  | |  | |  | |  | |  | | -.22 | | -.33 | | -.60 | | .43 | | .55 | | .77 | |
| 1. **Equal Treatment T1** |  | |  | |  | |  | |  | |  | |  | |  | |  | |  | |  | |  | |  | | .52 | | .50 | | -.35 | | -.22 | | -.20 | |
| 1. **Equal Treatment T2** |  | |  | |  | |  | |  | |  | |  | |  | |  | |  | |  | |  | |  | |  | | .66 | | -.17 | | -.40 | | -.30 | |
| 1. **Equal Treatment T3** |  | |  | |  | |  | |  | |  | |  | |  | |  | |  | |  | |  | |  | |  | |  | | -.28 | | -.39 | | -.57 | |
| 1. **Critical consciousness T1** |  | |  | |  | |  | |  | |  | |  | |  | |  | |  | |  | |  | |  | |  | |  | |  | | .52 | | .48 | |
| 1. **Critical consciousness T2** |  | |  | |  | |  | |  | |  | |  | |  | |  | |  | |  | |  | |  | |  | |  | |  | |  | | .63 | |
| 1. **Critical consciousness T3** |  |  | |  | |  | |  | |  | |  | |  | |  | |  | |  | |  | |  | |  | |  | |  | |  | |  | |  |

*Note.* The table shows Pearson's product moment correlation coefficient for sample sizes ranging from *N* = 755 – 955. Due to the large sample size, correlation values above 0.07-0.06 (i.e., trivial in effect size) were significant at *p* < 0.05; thus, for interpreting effects, we considered the strength of the association (namely Pearson's *r*) as an effect size.

# Online Resource 13. *Correlations main study variables and controls – Germany.*

|  | **2.** | **3.** | **4.** | **5.** | **6.** | **7.** | **8.** | **9.** | **10.** | **11.** | **12.** | **13.** | **14.** | **15.** | **16.** | **17.** | **18.** | **19.** |
| --- | --- | --- | --- | --- | --- | --- | --- | --- | --- | --- | --- | --- | --- | --- | --- | --- | --- | --- |
| 1. **Intervention** | .05 | .01 | .00 | -.13 | -.01 | -.02 | -.08 | .03 | -.09 | -.01 | .08 | -.04 | -.02 | .04 | .01 | .01 | -.04 | -.04 |
| 1. **Age** |  | -.08 | .14 | .11 | .15 | .15 | -.06 | .03 | .05 | .11 | .07 | -.03 | -.10 | -.10 | -.10 | .08 | .11 | .02 |
| 1. **Gender** |  |  | .00 | -.06 | -.02 | -.03 | -.04 | -.06 | -.05 | .04 | .03 | .03 | .00 | -.03 | .04 | .09 | .01 | .02 |
| 1. **Immigrant descent** |  |  |  | .27 | .25 | .30 | .37 | .37 | .37 | -.09 | -.09 | -.13 | -.13 | -.17 | -.10 | -.16 | -.16 | -.24 |
| 1. **Exploration T1** |  |  |  |  | .55 | .55 | .48 | .29 | .28 | .25 | .12 | .15 | -.20 | -.12 | -.09 | .14 | .08 | .03 |
| 1. **Exploration T2** |  |  |  |  |  | .66 | .29 | .46 | .29 | .18 | .16 | .18 | -.14 | -.17 | -.18 | .09 | .09 | .11 |
| 1. **Exploration T3** |  |  |  |  |  |  | .30 | .35 | .43 | .12 | .14 | .18 | -.17 | -.12 | -.18 | .05 | .10 | .12 |
| 1. **Resolution T1** |  |  |  |  |  |  |  | .48 | .55 | .10 | -.02 | .12 | -.17 | -.07 | -.13 | -.01 | -.11 | -.03 |
| 1. **Resolution T2** |  |  |  |  |  |  |  |  | .54 | .02 | .09 | .09 | -.12 | .03 | -.05 | -.05 | -.01 | -.03 |
| 1. **Resolution T3** |  |  |  |  |  |  |  |  |  | .08 | .14 | .13 | -.11 | .05 | -.03 | -.03 | .05 | .02 |
| 1. **Heritage Learning T1** |  |  |  |  |  |  |  |  |  |  | .46 | .42 | -.17 | .00 | .08 | .58 | .32 | .30 |
| 1. **Heritage Learning T2** |  |  |  |  |  |  |  |  |  |  |  | .66 | .05 | -.02 | .07 | .32 | .56 | .38 |
| 1. **Heritage Learning T3** |  |  |  |  |  |  |  |  |  |  |  |  | -.04 | -.02 | -.12 | .28 | .40 | .65 |
| 1. **Equal Treatment T1** |  |  |  |  |  |  |  |  |  |  |  |  |  | .31 | .26 | -.35 | -.07 | .01 |
| 1. **Equal Treatment T2** |  |  |  |  |  |  |  |  |  |  |  |  |  |  | .37 | -.12 | -.30 | -.02 |
| 1. **Equal Treatment T3** |  |  |  |  |  |  |  |  |  |  |  |  |  |  |  | -.06 | -.09 | -.31 |
| 1. **Critical consciousness T1** |  |  |  |  |  |  |  |  |  |  |  |  |  |  |  |  | .51 | .41 |
| 1. **Critical consciousness T2** |  |  |  |  |  |  |  |  |  |  |  |  |  |  |  |  |  | .55 |
| 1. **Critical consciousness T3** |  |  |  |  |  |  |  |  |  |  |  |  |  |  |  |  |  |  |

*Note.* The table shows Pearson's product moment correlation coefficient for sample sizes ranging from *N* = 326 – 583. Due to the large sample size, correlation values above 0.10-08 (i.e., small to trivial effect size) were significant at *p* < 0.05; thus, for interpreting effects, we considered the strength of the association (namely Pearson's *r*) as an effect size.

# Online Resource 14. *Significant (α = 0.05) differences between students with and without missing data, Italy and Germany.*

|  |  | **Students without missing data**  **(*N* = 718)** | **Students with missing data**  **(*N* = 237)** | **Overlapping area between distributions with and without missing data [95% - CI]** | **Phi**  **[95% - CI]** |
| --- | --- | --- | --- | --- | --- |
|  |  | ***M* (*SD*)** | ***M* (*SD*)** |  |  |
| **Italy** |  |  |  |  |  |
|  | Critical consciousness T3 | 2.44 (0.76) | 2.29 (0.64) | .86 [.80, .92] |  |
|  | Age | 15.06 (0.65) | 15.32 (0.75) | .77 [.70, .85] |  |
|  | Intervention | .51 (0.50) | .58 (0.49) |  | *φ* = 0.06 [0.00, 0.12] |
| **Germany** |  |  |  |  |  |
|  | Heritage cultural identity resolution T2 | 3.13 (0.83) | 3.37 (0.83) | .85 [.78, .92] |  |
|  | Heritage and intercultural learning T1 | 3.10 (0.91) | 2.70 (0.96) | .84 [.77, .91] |  |
|  | Heritage and intercultural learning T2 | 3.13 (0.84) | 2.91 (0.91) | .90 [.83, .96] |  |
|  | Heritage and intercultural learning T3 | 3.05 (0.96) | 2.73 (0.95) | .84 [.77, .91] |  |
|  | Critical consciousness T1 | 2.70 (0.99) | 2.36 (1.00) | .87 [.80, .94] |  |
|  | FASII | 4.53 (1.82) | 4.72 (1.97) | .95 [.90, .99] |  |
|  | Immigrant descent | 0.49 (0.50) | 0.63 (0.48) |  | *φ* = 0.14 [0.06, 0.23] |

*Note.* Only variables with significant (α = 0.05) differences between students with and without missing data, as well as variables that significantly (α = 0.05) predict attrition are represented in the table. For metric variables, overlapping area between distributions with and without missing data is reported - for example, for heritage and intercultural learning at T2, .90 indicates that 90% of the area of heritage and intercultural learning at T2 distributions for students with and without missing data overlap, indicating only minimal differences between both groups of students. For categorical variables, Phi is reported as effect size, with values closer to 0 indicating a weak association between missing data and the variable, and values closer to 1 indicating a strong association.

# Online Resource 15. *Model comparisons – Italian sample.*

|  |  | **Null model** | **Null model including random intercepts** | **Full model without stability coefficients** | **Full model** | **Full model including immigrant descent** | **Full model including immigrant descent and age** | **Final model** |
| --- | --- | --- | --- | --- | --- | --- | --- | --- |
| **Model** | **Heritage and intercultural learning T2 ~** | 1 | 1+ (1 \| class ID) | intervention +  (1 \| class ID) | heritage and intercultural learning T1 + intervention +  (1 \| class ID) | heritage and intercultural learning T1 + intervention + immigrant descent  + (1 \| class ID) | heritage and intercultural learning T1 + intervention + immigrant descent + age +  (1 \| class ID) | heritage and intercultural learning T1 + immigrant descent +  (1 \| class ID) |
|  | **Critical consciousness T2 ~** | 1 | 1+ (1 \| class ID) | intervention +  (1 \| class ID) | critical consciousness T1 + intervention +  (1 \| class ID) | critical consciousness T1 + intervention + immigrant descent +  (1 \| class ID) | critical consciousness T1 + intervention + immigrant descent + age +  (1 \| class ID) | critical consciousness T1 +  intervention +  (1 \| class ID) |
|  | **Equal treatment T2 ~** | 1 | 1+ (1 \| class ID) | intervention +  (1 \| class ID) | equal treatment T1 + intervention +  (1 \| class ID) | equal treatment T1 + intervention +  immigrant descent + (1 \| class ID) | equal treatment T1 + intervention +  immigrant descent + age +  (1 \| class ID) | equal treatment T1 +  (1 \| class ID) |
|  | **Heritage cultural identity exploration T2 ~** | 1 | 1+ (1 \| class ID) | critical consciousness T1 +  equal treatment T1 + heritage and intercultural learning T1 +  intervention +  intervention * critical consciousness T1 +  intervention * equal treatment T1 +  intervention *heritage and intercultural learning T1 +  (1 \| class ID) | heritage cultural identity exploration T1 + critical consciousness T1 +  equal treatment T1 +  heritage and intercultural learning T1 + intervention +  intervention * critical consciousness T1 +  intervention * equal treatment T1 +  intervention *heritage and intercultural learning T1 +  (1 \| class ID) | heritage cultural identity exploration T1 + critical consciousness T1 +  equal treatment T1 + heritage and intercultural learning T1 +  intervention +  intervention * critical consciousness T1 +  intervention * equal treatment T1 +  intervention *heritage and intercultural learning T1 +  immigrant descent + (1 \| class ID) | heritage cultural identity exploration T1 + critical consciousness T1 +  equal treatment T1 + heritage and intercultural learning T1 +  intervention +  intervention * critical consciousness T1 +  intervention * equal treatment T1 + intervention *heritage and intercultural learning T1 +  immigrant descent + age +  (1 \| class ID) | heritage cultural identity exploration T1 + critical consciousness T1 +  intervention +  intervention * critical consciousness T1 +  immigrant descent +  (1 \| class ID) |
|  | **Heritage cultural identity resolution T3 ~** | 1 | 1+ (1 \| class ID) | heritage cultural identity exploration T2 + critical consciousness T2 +  equal treatment T2 + heritage and intercultural learning T2 + intervention + intervention * critical consciousness T2 +  intervention * equal treatment T2 + intervention *heritage and intercultural learning T2 +  (1 \| class ID) | heritage cultural identity resolution T1 + heritage cultural identity exploration T2 +  critical consciousness T2 +  equal treatment T2 +  heritage and intercultural learning T2 +  intervention +  intervention * critical consciousness T2 +  intervention * equal treatment T2 +  intervention *heritage and intercultural learning T2 +  (1 \| class ID) | heritage cultural identity resolution T1 + heritage cultural identity exploration T2 +  critical consciousness T2 +  equal treatment T2 +  heritage and intercultural learning T2 +  intervention +  intervention * critical consciousness T2 +  intervention * equal treatment T2 + intervention *heritage and intercultural learning T2 +  immigrant descent + (1 \| class ID) | heritage cultural identity resolution T1 + heritage cultural identity exploration T2 + critical consciousness T2 +  equal treatment T2 + heritage and intercultural learning T2 +  intervention +  intervention * critical consciousness T2 +  intervention * equal treatment T2 + intervention *heritage and intercultural learning T2 +  immigrant descent + age +  (1 \| class ID) | heritage cultural identity resolution T1 + heritage cultural identity exploration T2 + critical consciousness T2 +  heritage and intercultural learning T2 + intervention +  intervention* heritage & intercultural learning T2 + immigrant descent +  (1 \| class ID) |
| **Bayesian R²** | **Heritage and intercultural learning T2 ~** | .00 | .01 | .02 | .27 | .27 | .28 | .27 |
|  | **Critical consciousness T2 ~** | .00 | .03 | .03 | .24 | .24 | .24 | .24 |
|  | **Equal treatment T2 ~** | .00 | .12 | .12 | .28 | .28 | .28 | .28 |
|  | **Heritage cultural identity exploration T2 ~** | .00 | .02 | .04 | .38 | .38 | .38 | .38 |
|  | **Heritage cultural identity resolution T3 ~** | .00 | .02 | .43 | .44 | .44 | .44 | .43 |
| **Loo model weights** |  | .000 | .000 | .000 | .000 | .001 | .000 | .998 |

*Note.* The table shows an overview of theoretically informed multilevel models that were run and compared. Each row shows one dependent variable (same for each model), with each column indicating the predictors specified for each model. The full model including immigrant descent and age was reduced step-by-step, until LOO indicated that the most likely model to describe the data was found (=final model). Bayesian R² shows the proportion of variance explained by the defined model in each level of the multilevel model. Loo (leave-one-out cross validation) model weights show likelihood of the model compared to other models.

| Online Resource 16. *Model comparisons – German sample.* | | | | | | | | | |
| --- | --- | --- | --- | --- | --- | --- | --- | --- | --- |
|  |  | **Null model** | **Null model including random intercepts** | **Full model without stability coefficients** | **Full model** | **Full model including immigrant descent** | **Full model including immigrant descent and FASII** | **Final model** |  |
| **Model** | **Heritage and intercultural learning T2 ~** | 1 | 1+ (1 \| class ID) | intervention +  (1 \| class ID) | heritage and intercultural learning T1 + intervention +  (1 \| class ID) | heritage and intercultural learning T1 +  intervention +  immigrant descent + (1 \| class ID) | heritage and intercultural learning T1 +  intervention +  immigrant descent + FASII +  (1 \| class ID) | heritage and intercultural learning T1 + intervention +  (1 \| class ID) |  |
|  | **Critical consciousness T2 ~** | 1 | 1+ (1 \| class ID) | intervention +  (1 \| class ID) | critical consciousness T1 +  intervention +  (1 \| class ID) | critical consciousness T1 +  intervention +  immigrant descent + (1 \| class ID) | critical consciousness T1 +  intervention +  immigrant descent + FASII +  (1 \| class ID) | critical consciousness T1 +  intervention +  immigrant descent + (1 \| class ID) |  |
|  | **Equal treatment T2 ~** | 1 | 1+ (1 \| class ID) | intervention +  (1 \| class ID) | equal treatment T1 +  intervention +  (1 \| class ID) | equal treatment T1 + intervention +  immigrant descent + (1 \| class ID) | equal treatment T1 + intervention +  immigrant descent + FASII +  (1 \| class ID) | equal treatment T1 + intervention +  immigrant descent + (1 \| class ID) |  |
|  | **Heritage cultural identity exploration T2 ~** | 1 | 1+ (1 \| class ID) | critical consciousness T1 +  equal treatment T1 + heritage and intercultural learning T1 +  intervention +  intervention * critical consciousness T1 +  intervention * equal treatment T1 + intervention *heritage and intercultural learning T1 +  (1 \| class ID) | heritage cultural identity exploration T1 + critical consciousness T1 +  equal treatment T1 + heritage and intercultural learning T1 + intervention +  intervention * critical consciousness T1 +  intervention * equal treatment T1 +  intervention *heritage and intercultural learning T1 + (1 \| class ID) | heritage cultural identity exploration T1 + critical consciousness T1 +  equal treatment T1 + heritage and intercultural learning T1 +  intervention +  intervention * critical consciousness T1 +  intervention * equal treatment T1 + intervention *heritage and intercultural learning T1 +  immigrant descent + (1 \| class ID) | heritage cultural identity exploration T1 + critical consciousness T1 +  equal treatment T1 + heritage and intercultural learning T1 +  intervention +  intervention * critical consciousness T1 +  intervention * equal treatment T1 + intervention *heritage and intercultural learning T1 +  immigrant descent + FASII +  (1 \| class ID) | heritage cultural identity exploration T1 + critical consciousness T1 +  equal treatment T1 + intervention +  immigrant descent  + (1 \| class_id) |  |
|  | **Heritage cultural identity resolution T3 ~** | 1 | 1+ (1 \| class ID) | heritage cultural identity exploration T2 + critical consciousness T2 +  equal treatment T2 + heritage and intercultural learning T2 +  intervention +  intervention * critical consciousness T2 +  intervention * equal treatment T2 + intervention *heritage and intercultural learning T2 +  (1 \| class ID) | heritage cultural identity resolution T1 + heritage cultural identity exploration T2 +  critical consciousness T2 +  equal treatment T2 +  heritage and intercultural learning T2 + intervention +  intervention * critical consciousness T2 +  intervention * equal treatment T2 +  intervention *heritage and intercultural learning T2 + (1 \| class ID) | heritage cultural identity resolution T1 + heritage cultural identity exploration T2 +  critical consciousness T2 +  equal treatment T2 + heritage and intercultural learning T2 +  intervention +  intervention * critical consciousness T2 +  intervention * equal treatment T2 + intervention *heritage and intercultural learning T2 +  immigrant descent + (1 \| class ID) | heritage cultural identity resolution T1 + heritage cultural identity exploration T2 +  critical consciousness T2 +  equal treatment T2 + heritage and intercultural learning T2 +  intervention +  intervention * critical consciousness T2 +  intervention * equal treatment T2 + intervention *heritage and intercultural learning T2 +  immigrant descent + FASII +  (1 \| class ID) | heritage cultural identity resolution T1 + equal treatment T2 + immigrant descent + (1 \| class_id) |  |
| **Bayesian R²** | **Heritage and intercultural learning T2 ~** | .00 | .09 | .09 | .24 | .25 | .25 | .24 |  |
|  | **Critical consciousness T2 ~** | .00 | .06 | .06 | .27 | .28 | .28 | .27 |  |
|  | **Equal treatment T2 ~** | .00 | .04 | .05 | .16 | .16 | .16 | .16 |  |
|  | **Heritage cultural identity exploration T2 ~** | .00 | .05 | .09 | .31 | .34 | .34 | .34 |  |
|  | **Heritage cultural identity resolution T3 ~** | .00 | .09 | .27 | .36 | .39 | .39 | .33 |  |
| **Loo model weights** |  | .000 | .000 | .000 | .000 | .005 | .000 | .995 |  |

*Note.* The table shows an overview of theoretically informed multilevel models that were run and compared. Each row shows one dependent variable (same for each model), with each column indicating the predictors specified for each model. The full model including immigrant descent and FASII was reduced step-by-step, until LOO indicated that the most likely model to describe the data was found (=final model). Bayesian R² shows the proportion of variance explained by the defined model in each level of the multilevel model. Loo (leave-one-out cross validation) model weights show likelihood of the model compared to other models.

# Online Resource 17. *Probability of effect prior and post – final model Italy.*

| **Dependent variable** | **Independent variable** | **Probability of effect** | | | |
| --- | --- | --- | --- | --- | --- |
|  |  | **Prior** | | **Post** | |
|  |  | **Below ROPE -.1** | **Above ROPE .1** | **Below ROPE -.1** | **Above ROPE .1** |
| Heritage and intercultural learning T2 |  |  |  |  |  |
|  | heritage and intercultural learning T1 | .01 | .93 | .00 | 1.00 |
|  | immigrant descent | .43 | .43 | .05 | .00 |
| Critical consciousness T2 |  |  |  |  |  |
|  | critical consciousness T1 | .01 | .93 | .00 | 1.00 |
|  | intervention | .24 | .64 | .00 | .25 |
| Equal treatment T2 |  |  |  |  |  |
|  | equal treatment T1 | .01 | .93 | .00 | 1.00 |
| Heritage cultural identity exploration T2 |  |  |  |  |  |
|  | heritage cultural identity exploration T1 | .01 | .93 | .00 | 1.00 |
|  | critical consciousness T1 | .24 | .64 | .00 | .08 |
|  | intervention | .01 | .93 | .00 | .95 |
|  | intervention * critical consciousness T1 | .24 | .64 | .33 | .00 |
|  | immigrant descent | .01 | .93 | .00 | .56 |
| Heritage cultural identity resolution T3 |  |  |  |  |  |
|  | heritage cultural identity resolution T1 | .01 | .93 | .00 | 1.00 |
|  | heritage cultural identity exploration T2 | .24 | .64 | .00 | 1.00 |
|  | critical consciousness T2 | .24 | .64 | .29 | .01 |
|  | heritage and intercultural learning T2 | .24 | .64 | .00 | .80 |
|  | intervention | .24 | .64 | .00 | .98 |
|  | intervention* heritage & intercultural learning T2 | .24 | .64 | .82 | .00 |
|  | immigrant descent | .01 | .93 | .00 | .32 |

*Note.* The table shows the regions of practical equivalence (ROPE; Kruschke, 2014), which defines values that are equivalent to the null effect. The ROPE was set from −0.1 to + 0.1 for all model parameters. Values within this region are defined to be equal to the null effect. The lower the percentage of overlap between the ROPE and the highest posterior density interval, the stronger the support for the investigated effect. The prior column indicates the expected probability that values will be outside of the ROPE as defined by theoretical priors. The post column indicated the percentage of values outside of the ROPE as indicated by the estimated model parameters of the final Italian model.

# Online Resource 18. *Probability of effect prior and post – final model Germany.*

| **Dependent variable** | **Independent variable** | **Probability of effect** | | | |
| --- | --- | --- | --- | --- | --- |
|  |  | **Prior** | | **Post** | |
|  |  | **Below ROPE -.1** | **Above ROPE .1** | **Below ROPE -.1** | **Above ROPE .1** |
| Heritage and intercultural learning T2 |  |  |  |  |  |
|  | heritage and intercultural learning T1 | .00 | 1.00 | .00 | 1.00 |
|  | intervention | .00 | .09 | .00 | .12 |
| Critical consciousness T2 |  |  |  |  |  |
|  | critical consciousness T1 | .00 | 1.00 | .00 | 1.00 |
|  | intervention | .03 | .44 | .01 | .24 |
|  | immigrant descent | .30 | .02 | .42 | .02 |
| Equal treatment T2 |  |  |  |  |  |
|  | equal treatment T1 | .00 | 1.00 | .00 | 1.00 |
|  | intervention | .38 | .03 | .23 | .01 |
|  | immigrant descent | .05 | .10 | .76 | .00 |
| Heritage cultural identity exploration T2 |  |  |  |  |  |
|  | heritage cultural identity exploration T1 | .00 | 1.00 | .00 | 1.00 |
|  | critical consciousness T1 | .02 | .12 | .00 | .03 |
|  | equal treatment T1 | .04 | .04 | .06 | .00 |
|  | intervention | .06 | .87 | .00 | .98 |
|  | immigrant descent | .01 | .50 | .00 | .99 |
| Heritage cultural identity resolution T3 |  |  |  |  |  |
|  | heritage cultural identity resolution T1 | .00 | 1.00 | .00 | 1.00 |
|  | equal treatment T2 | .08 | .12 | .03 | .12 |
|  | immigrant descent | .01 | .33 | .00 | 1.00 |

*Note.* The table shows the regions of practical equivalence (ROPE; Kruschke, 2014), which defines values that are equivalent to the null effect. The ROPE was set from −0.1 to + 0.1 for all model parameters. Values within this region are defined to be equal to the null effect. The lower the percentage of overlap between the ROPE and the highest posterior density interval, the stronger the support for the investigated effect. The prior column indicates the expected probability that values will be outside of the ROPE as defined by priors based on the Italian posteriors. The post column indicated the percentage of values outside of the ROPE as indicated by the estimated model parameters of the final German model.

# Online Resource 19. *Robustness check comparing list-wise deletion and imputed data - Italian sample.*

| **Dependent variable** | **Independent variable** | **Coefficients** | | **Bayesian R2** | |
| --- | --- | --- | --- | --- | --- |
|  |  | **List-wise deletion**  **(*N* = 718)** | **Imputation**  **(*N* = 955)** | **List-wise deletion**  **(*N* = 718)** | **Imputation**  **(*N* = 955)** |
| Heritage and intercultural learning T2 |  |  |  | .27 | .26 |
|  | heritage and intercultural learning T1 | .41 [.36, .46] | .40 [.35, .44] |  |  |
|  | immigrant descent | -.04 [-.11, .02] | -.06 [-.12, .00] |  |  |
|  | (1 \| class ID) | .04 [.00, .10] | .04 [.00, .09] |  |  |
| Critical consciousness T2 |  |  |  | .24 | .22 |
|  | critical consciousness T1 | .48 [.42, .54] | .46 [.41, .51] |  |  |
|  | intervention | .07 [-.03, .17] | .04 [-.05, .14] |  |  |
|  | (1 \| class ID) | .11 [.03, .17] | .09 [.03, .15] |  |  |
| Equal treatment T2 |  |  |  | .28 | .25 |
|  | equal treatment T1 | .51 [.44, .59] | .50 [.44, .57] |  |  |
|  | (1 \| class ID) | .15 [.08, .22] | .14 [.08, .21] |  |  |
| Heritage cultural identity exploration T2 |  |  |  | .38 | .37 |
|  | heritage cultural identity exploration T1 | .52 [.47, .57] | .54 [.49, .59] |  |  |
|  | critical consciousness T1 | .05 [-.01., .12] | .05 [-.02., .11] |  |  |
|  | intervention | .28 [.06, .52] | .18 [-.04, .40] |  |  |
|  | intervention * critical consciousness T1 | -.08 [-.17, .01] | -.04 [-.12, .05] |  |  |
|  | immigrant descent | .10 [.03, .17] | .07 [.00, .13] |  |  |
|  | (1 \| class ID) | .07 [.01, .12] | .06 [.01, .11] |  |  |
| Heritage cultural identity resolution T3 |  |  |  | .43 | .41 |
|  | heritage cultural identity resolution T1 | .40 [.34, .46] | .40 [.34, .46] |  |  |
|  | heritage cultural identity exploration T2 | .37 [.24, .50] | .40 [.28, .52] |  |  |
|  | critical consciousness T2 | -.06 [-.20, .07] | -.08 [-.22, .06] |  |  |
|  | heritage and intercultural learning T2 | .17 [.01, .34] | .13 [-.02, .29] |  |  |
|  | intervention | .44 [.12, .75] | .39 [.10, .69] |  |  |
|  | intervention* heritage & intercultural learning T2 | -.15 [-.27, -.04] | -.13 [-.23, -.02] |  |  |
|  | immigrant descent | .08 [-.01, .17] | .06 [-.02, .14] |  |  |
|  | (1 \| class ID) | .10 [.02, .16] | .07 [.01, .13] |  |  |

*Note.* The table shows difference in model coefficients the data with list-wise deletion and imputation. Most differences are minimal (between .02 and .03). However, the effect of intervention and immigrant descent on heritage cultural identity exploration at T2, and the effect of heritage and intercultural learning on T2 on heritage cultural identity resolution at T3 change to include 0 for the imputed data. The Bayesian R² also shows minimal differences (between .01 and .03). Repeating comparisons of LOO weights show, that also for imputed data the best model is over 997 times more likely than the other models.

# Online Resource 20*. Robustness check comparing list-wise deletion and imputed data - German sample.*

| **Dependent variable** | **Independent variable** | **Coefficients** | | **Bayesian R2** | |
| --- | --- | --- | --- | --- | --- |
|  |  | **List-wise deletion**  **(*N* = 274)** | **Imputation**  **(*N* = 583)** | **List-wise deletion**  **(*N* = 274)** | **Imputation**  **(*N* = 583)** |
| Heritage and intercultural learning T2 |  |  |  | .24 | .23 |
|  | heritage and intercultural learning T1 | .39 [.33, .43] | .40 [.34, .43] |  |  |
|  | intervention | .05 [-.04, .15] | .13 [.02, .26] |  |  |
|  | (1 \| class ID) | .19 [.05, .35] | .13 [.02, .24] |  |  |
| Critical consciousness T2 |  |  |  | .27 | .25 |
|  | critical consciousness T1 | .46 [.40, .51] | .44 [.39, .49] |  |  |
|  | intervention | .06 [-.06, .17] | .00 [-.14, .10] |  |  |
|  | immigrant descent | -.08 [-.26, .10] | -.07 [-.21, .07] |  |  |
|  | (1 \| class ID) | .10 [.01, .24] | .13 [.01, .25] |  |  |
| Equal treatment T2 |  |  |  | .16 | .13 |
|  | equal treatment T1 | .34 [.22, .46] | .34 [.24, .44] |  |  |
|  | intervention | -.06 [-.17, .07] | .08 [-.04, .20] |  |  |
|  | immigrant descent | -.17 [-.38, .03] | -.16 [-.32, .00] |  |  |
|  | (1 \| class ID) | .12 [.01, .29] | .07 [.01, .23] |  |  |
| Heritage cultural identity exploration T2 |  |  |  | .34 | .31 |
|  | heritage cultural identity exploration T1 | .53 [.47, .59] | .53 [.47, .58] |  |  |
|  | critical consciousness T1 | .04 [-.03, .10] | .05 [-.00, .10] |  |  |
|  | equal treatment T1 | -.03 [-.12, .04] | -.04 [-.11, .02] |  |  |
|  | intervention | .30 [.10, .51] | .18 [.02, .33] |  |  |
|  | immigrant descent | .31 [.15, .47] | .27 [.13, .42] |  |  |
|  | (1 \| class ID) | .09 [.00, .25] | .10 [.01, .23] |  |  |
| Heritage cultural identity resolution T3 |  |  |  | .33 | .32 |
|  | heritage cultural identity resolution T1 | .43 [.37, .51] | .45 [.39, .54] |  |  |
|  | equal treatment T2 | .02 [-.11, .17] | -.00 [-.12, .15] |  |  |
|  | immigrant descent | .36 [.22, .52] | .33 [.22, .45] |  |  |
|  | (1 \| class ID) | .14 [.01, .31] | .08 [.00, .19] |  |  |

*Note.* The table shows difference in model coefficients the data with list-wise deletion and imputation. Differences are larger than within the Italian models (between .00 and .12). However, only one coefficient changed to include or not include 0: in the model with list-wise deletion the CI of intervention on heritage and intercultural learning at T2 included 0 (*β* = .05 [-.04, .15]), while in the imputed model the intervention had a positive effect on heritage intercultural learning at T2 (*β* = .13 [.02, .26]). The Bayesian R² also shows minimal differences (between .01 and .03). Repeating comparisons of LOO weights show, that also for imputed data the best model is over 999 times more likely than the other models.

# Online Resource 21. *Robustness check with additional covariates – Italian sample.*

| **Dependent variable** | **Independent variable** | **Coefficients** | | **Bayesian R2** | |
| --- | --- | --- | --- | --- | --- |
|  |  | **Final model**  **(*N* = 718)** | **Additional covariates**  **(*N* = 689)** | **Final model**  **(*N* = 718)** | **Additional covariates**  **(*N* = 689)** |
| Heritage and intercultural learning T2 |  |  |  | .27 | .28 |
|  | heritage and intercultural learning T1 | .41 [.36, .46] | .40 [.35, .45] |  |  |
|  | immigrant descent | -.04 [-.11, .02] | -.21 [-.83, .38] |  |  |
|  | first-generation immigrant | - | .29 [-.50, .73] |  |  |
|  | second-generation immigrant | - | .11 [-.31, .93] |  |  |
|  | Percentage of students of immigrant descent | - | -.00 [-.00, .00] |  |  |
|  | Technical track | - | -.06 [-.16, .05] |  |  |
|  | Academic track | - | .13 [-.00, .26] |  |  |
|  | Gender | - | -.01 [-.08, .06] |  |  |
|  | (1 \| class ID) | .04 [.00, .10] | .04 [.00, .09] |  |  |
| Critical consciousness T2 |  |  |  | .24 | .24 |
|  | critical consciousness T1 | .48 [.42, .54] | .47 [.40, .53] |  |  |
|  | intervention | .07 [-.03, .17] | .04 [-.06, .15] |  |  |
|  | first-generation immigrant | - | -.04 [-.23, .15] |  |  |
|  | second-generation immigrant | - | -.06 [-.18, .07] |  |  |
|  | Percentage of students of immigrant descent | - | -.00 [-.01, .00] |  |  |
|  | Technical track | - | -.17 [-.31, -.02] |  |  |
|  | Academic track | - | -.06 [-.24, .13] |  |  |
|  | Gender | - | -.11 [-.21, -.01] |  |  |
|  | (1 \| class ID) | .11 [.03, .17] | .09 [.02, .16] |  |  |
| Equal treatment T2 |  |  |  | .28 | .31 |
|  | equal treatment T1 | .51 [.44, .59] | .50 [.43, .58] |  |  |
|  | first-generation immigrant | - | .06 [-.13, .25] |  |  |
|  | second-generation immigrant | - | .03 [-.08, .16] |  |  |
|  | Percentage of students of immigrant descent | - | -.00 [-.01, .00] |  |  |
|  | Technical track | - | .18 [.03, .32] |  |  |
|  | Academic track | - | .17 [-.02, .37] |  |  |
|  | Gender | - | .28 [.17, .38] |  |  |
|  | (1 \| class ID) | .15 [.08, .22] | .09 [.01, .18] |  |  |
| Heritage cultural identity exploration T2 |  |  |  | .38 | .38 |
|  | heritage cultural identity exploration T1 | .52 [.47, .57] | .52 [.46, .57] |  |  |
|  | critical consciousness T1 | .05 [-.01., .12] | .06 [-.01, .12] |  |  |
|  | intervention | .28 [.06, .52] | .26 [.03, .48] |  |  |
|  | intervention * critical consciousness T1 | -.08 [-.17, .01] | -.06 [-.15, .03] |  |  |
|  | immigrant descent | .10 [.03, .17] | .23 [-.11, .49] |  |  |
|  | first-generation immigrant | - | -.12 [-.39, .24] |  |  |
|  | second-generation immigrant | - | -.14 [-.40, .21] |  |  |
|  | Percentage of students of immigrant descent | - | .00 [-.00, .00] |  |  |
|  | Technical track | - | .00 [-.10, .12] |  |  |
|  | Academic track | - | -.02 [-.15, .12] |  |  |
|  | Gender | - | .02 [-.05, .09] |  |  |
|  | (1 \| class ID) | .07 [.01, .12] | .07 [.01, .13] |  |  |
| Heritage cultural identity resolution T3 |  |  |  | .43 | .44 |
|  | heritage cultural identity resolution T1 | .40 [.34, .46] | .36 [.30, .43] |  |  |
|  | heritage cultural identity exploration T2 | .37 [.24, .50] | .40 [.26, .53] |  |  |
|  | critical consciousness T2 | -.06 [-.20, .07] | -.05 [-.20, .09] |  |  |
|  | heritage and intercultural learning T2 | .17 [.01, .34] | .18 [.01, .34] |  |  |
|  | intervention | .44 [.12, .75] | .43 [.11, .75] |  |  |
|  | intervention* heritage & intercultural learning T2 | -.15 [-.27, -.04] |  |  |  |
|  | immigrant descent | .08 [-.01, .17] | .22 [-.21, .48] |  |  |
|  | first-generation immigrant | - | -.13 [-.43, .31] |  |  |
|  | second-generation immigrant | - | -.15 [-.44, .28] |  |  |
|  | Percentage of students of immigrant descent | - | .00 [-.00, .01] |  |  |
|  | Technical track | - | .11 [-.02, .24] |  |  |
|  | Academic track | - | .09 [-.08, .25] |  |  |
|  | Gender | - | -.03 [-.11, .05] |  |  |
|  | (1 \| class ID) | .10 [.02, .16] | .10 [.02, .16] |  |  |

*Note.* The table shows differences in model coefficients between the final model and a model with additional covariates. Priors were selected to align with the assumption of no group differences, employing coefficients from a student t-distribution with 3 degrees of freedom, centered at 0, and a scale parameter of 0.5. First (1) and second (2) immigrant generations were coded against students of non-immigrant descent (0). Technical (1) and academic (2) tracks were coded against vocational (0) school tracks. Gender was coded as male (0) and female (1). No covariate indicating the covid-19 pandemic was added in the Italian sample, as all data was collected during the covid-19 pandemic, in the school year 2021-22. No coefficient changed to include or not include 0 by including the additional covariates. Technical track and gender showed an effect on critical consciousness and equal treatment at T2. The Bayesian R² also shows minimal differences (between .01 and .04). Repeating comparisons of LOO weights show, that the final model is 991 times more likely than the model with the additional covariates.

# Online Resource 22. *Robustness check with additional covariates - German sample.*

| **Dependent variable** | **Independent variable** | **Coefficients** | | **Bayesian R2** | |
| --- | --- | --- | --- | --- | --- |
|  |  | **Final model**  **(*N* = 274)** | **Additional covariates**  **(*N* = 267)** | **List-wise deletion**  **(*N* = 274)** | **Additional covariates**  **(*N* = 267)** |
| Heritage and intercultural learning T2 |  |  |  | .24 | .28 |
|  | heritage and intercultural learning T1 | .39 [.33, .43] | .38 [.30, .43] |  |  |
|  | intervention | .05 [-.04, .15] | .06 [-.04, .17] |  |  |
|  | first-generation immigrant | - | .16 [-.11, .45] |  |  |
|  | second-generation immigrant | - | -.10 [-.34, .17] |  |  |
|  | Percentage of students of immigrant descent | - | .00 [-.01, .01] |  |  |
|  | *Realschule* | - | -.23 [-.57, .15] |  |  |
|  | *Gesamtschule* | - | -.20 [-.72, .31] |  |  |
|  | Pre vs. during covid-19 | - | .16 [-.30, .62] |  |  |
|  | Gender | - | -.05 [-.22, .13] |  |  |
|  | (1 \| class ID) | .19 [.05, .35] | .16 [.01, .33] |  |  |
| Critical consciousness T2 |  |  |  | .27 | .28 |
|  | critical consciousness T1 | .46 [.40, .51] | .44 [.39, .50] |  |  |
|  | intervention | .06 [-.06, .17] | .04 [-.08, .14] |  |  |
|  | immigrant descent | -.08 [-.26, .10] | .00 [-.60, .62] |  |  |
|  | first-generation immigrant | - | .03 [-.56, .65] |  |  |
|  | second-generation immigrant | - | -.00 [-.62, .61] |  |  |
|  | Percentage of students of immigrant descent | - | -.00 [-.01, .00] |  |  |
|  | *Realschule* | - | -.12 [-.44, .20] |  |  |
|  | *Gesamtschule* | - | .05 [-.45, .59] |  |  |
|  | Pre vs. during covid-19 | - | .29 [-.14, .75] |  |  |
|  | Gender | - | -.07 [-.27, .12] |  |  |
|  | (1 \| class ID) | .10 [.01, .24] | .09 [.00, .23] |  |  |
| Equal treatment T2 |  |  |  | .16 | .19 |
|  | equal treatment T1 | .34 [.22, .46] | .37 [.25, .48] |  |  |
|  | intervention | -.06 [-.17, .07] | .07 [-.06, .18] |  |  |
|  | immigrant descent | -.17 [-.38, .03] | -.04 [-.64, .57] |  |  |
|  | first-generation immigrant | - | -.02 [-.63, .60] |  |  |
|  | second-generation immigrant | - | -.03 [-.65, .60] |  |  |
|  | Percentage of students of immigrant descent | - | -.00 [-.01, .00] |  |  |
|  | *Realschule* | - | -.00 [-.34, .33] |  |  |
|  | *Gesamtschule* | - | -.06 [-.57, 45] |  |  |
|  | Pre vs. during covid-19 | - | -.26 [-.72, .19] |  |  |
|  | Gender | - | -.08 [-.27, .12] |  |  |
|  | (1 \| class ID) | .12 [.01, .29] | .09 [.00, .26] |  |  |
| Heritage cultural identity exploration T2 |  |  |  | .34 | .36 |
|  | heritage cultural identity exploration T1 | .53 [.47, .59] | .53 [.47, .59] |  |  |
|  | critical consciousness T1 | .04 [-.03, .10] | .04 [-.02, .10] |  |  |
|  | equal treatment T1 | -.03 [-.12, .04] | -.04 [-.12, .03] |  |  |
|  | intervention | .30 [.10, .51] | .26 [.09, .43] |  |  |
|  | immigrant descent | .31 [.15, .47] | .29 [-.00, .54] |  |  |
|  | first-generation immigrant | - | .15 [-.24, .54] |  |  |
|  | second-generation immigrant | - | -.32 [-.67, .06] |  |  |
|  | Percentage of students of immigrant descent | - | .00 [-.00, .01] |  |  |
|  | *Realschule* | - | -.31 [-.65, .02] |  |  |
|  | *Gesamtschule* | - | .25 [-.25, .78] |  |  |
|  | Pre vs. during covid-19 | - | .02 [-.40, .47] |  |  |
|  | Gender | - | -.05 [-.25, .14] |  |  |
|  | (1 \| class ID) | .09 [.00, .25] | .08 [.00, .21] |  |  |
| Heritage cultural identity resolution T3 |  |  |  | .33 | .37 |
|  | heritage cultural identity resolution T1 | .43 [.37, .51] | .41 [.35, .49] |  |  |
|  | equal treatment T2 | .02 [-.11, .17] | -.03 [-.16, .11] |  |  |
|  | immigrant descent | .36 [.22, .52] | .33 [.07, .64] |  |  |
|  | first-generation immigrant | - | .28 [-.10, .64] |  |  |
|  | second-generation immigrant | - | .06 [-.30, .40] |  |  |
|  | Percentage of students of immigrant descent | - | .00 [-.01, .01] |  |  |
|  | *Realschule* | - | -.25 [-.55, .07] |  |  |
|  | *Gesamtschule* | - | -.36 [-.85, .13] |  |  |
|  | Pre vs. during covid-19 | - | -.16 [-.57, .25] |  |  |
|  | Gender | - | -.15 [-.32, .02] |  |  |
|  | (1 \| class ID) | .14 [.01, .31] | .11 [.01, .29] |  |  |

*Note.* The table shows differences in model coefficients between the final model and a model with additional covariates. Priors were selected to align with the assumption of no group differences, employing coefficients from a student t-distribution with 3 degrees of freedom, centered at 0, and a scale parameter of 0.5. First (1) and second (1) immigrant generations were coded against students of non-immigrant descent (0). School type was coded as *Realschule* (1), *Gesamtschule* (2) versus *Gymnasium* (0). The pandemic variable was coded as pre-COVID-19 (0) versus during-COVID-19 (1). Gender was coded as male (0) and female (1). No coefficient changed to include or not include 0 by including the additional covariates, and no additional covariates showed an effect on a main study variable. The Bayesian R² also shows minimal differences (between .01 and .04). Repeating comparisons of LOO weights show, that the final model is 990 times more likely than the model with the additional covariates.
